# Challenges in analysis and interpretation of microsatellite data for population genetic studies

**DOI:** 10.1002/ece3.1305

**Published:** 2014-10-30

**Authors:** Alexander I Putman, Ignazio Carbone

**Affiliations:** Department of Plant Pathology, North Carolina State UniversityRaleigh, North Carolina, 27695-7616

**Keywords:** Data analysis, microsatellite, population genetics, population structure

## Abstract

Advancing technologies have facilitated the ever-widening application of genetic markers such as microsatellites into new systems and research questions in biology. In light of the data and experience accumulated from several years of using microsatellites, we present here a literature review that synthesizes the limitations of microsatellites in population genetic studies. With a focus on population structure, we review the widely used fixation (*F*_ST_) statistics and Bayesian clustering algorithms and find that the former can be confusing and problematic for microsatellites and that the latter may be confounded by complex population models and lack power in certain cases. Clustering, multivariate analyses, and diversity-based statistics are increasingly being applied to infer population structure, but in some instances these methods lack formalization with microsatellites. Migration-specific methods perform well only under narrow constraints. We also examine the use of microsatellites for inferring effective population size, changes in population size, and deeper demographic history, and find that these methods are untested and/or highly context-dependent. Overall, each method possesses important weaknesses for use with microsatellites, and there are significant constraints on inferences commonly made using microsatellite markers in the areas of population structure, admixture, and effective population size. To ameliorate and better understand these constraints, researchers are encouraged to analyze simulated datasets both prior to and following data collection and analysis, the latter of which is formalized within the approximate Bayesian computation framework. We also examine trends in the literature and show that microsatellites continue to be widely used, especially in non-human subject areas. This review assists with study design and molecular marker selection, facilitates sound interpretation of microsatellite data while fostering respect for their practical limitations, and identifies lessons that could be applied toward emerging markers and high-throughput technologies in population genetics.

## Introduction

Technological improvements have greatly expanded the realm of genetic markers in biological research, resulting in the ability to more efficiently collect datasets of ever-increasing size and, for example, address more questions in the context of population genetics. Therefore, it is becoming increasingly necessary to focus more attention on understanding the practical limitations of various analyses and applying increased caution when interpreting results (Karl et al. 2012). Such issues without a focus on marker type have been partly addressed in previous reviews with respect to statistical methods (Marjoram and Tavaré 2006), programs (Excoffier and Heckel 2006), and polyploid organisms (Dufresne et al. 2014; Meirmans and Van Tienderen 2012; Wang and Scribner 2014) in population genetics.

Also known as simple sequence repeats (SSRs) or short tandem repeats (STRs), microsatellites are tandemly repeating units of DNA 1 or 2–6 bp in length that are widely distributed throughout the nuclear genomes of eukaryotes (Bhargava and Fuentes 2010). Because they are highly polymorphic, microsatellites are desired for use as genetic markers for purposes that include fingerprinting, parentage identification, genetic mapping, conservation, and population genetics (Buschiazzo and Gemmell 2006; Chistiakov et al. 2006; Bhargava and Fuentes 2010; Guichoux et al. 2011). The recent history of expanding use of microsatellites in research has been greatly assisted by the availability of refined methods of marker development, genotyping methods, and data scoring (Glenn and Schable 2005; Selkoe and Toonen 2006; Gardner et al. 2011; Guichoux et al. 2011; Kelly et al. 2011; Campagne et al. 2012; Hess et al. 2012).

### Properties of microsatellites

While microsatellites are widely employed as markers in population studies, some of the properties that make microsatellites desirable as markers may also confound population genetic inference. One of the most significant problems associated with population genetic inferences using microsatellites is their mechanism of mutation. In general, inference and predictions on the forces influencing populations require the modeling of the mutational process generating genetic diversity. Much of classical population genetics theory is based on the infinite allele model (IAM) for allozyme data or the infinite sites model of DNA substitution mutation (Tajima 1996). In these theoretical models, each mutation event results in a new, unique allele, and mutation at a given locus is assumed to occur only once. Nucleotide substitutions detected by gene fragment sequencing, and more recently single-nucleotide polymorphisms (SNPs), can be analyzed using one of these models (Tajima 1996). In contrast to DNA substitutions, microsatellites are believed to primarily mutate by strand slippage during DNA replication, which manifests as the gain or loss of repeat unit(s). In general, the IAM is a poor descriptor of this process because new alleles do not arise independently of the previous allele (i.e., mutations have a history) (Goldstein et al. 1995; Slatkin 1995; Bhargava and Fuentes 2010). The stepwise mutation model (SMM), in which each mutational event results in the gain or loss of a single repeat unit, is a more appropriate theoretical description of the microsatellite mutation process (Slatkin 1995). In practice, however, the mutation model best describing microsatellite evolution varies among loci, and the behavior of a given locus can be described as falling on a range bordered by the IAM at one end and SMM at the other (Piry et al. 1999). Mutational processes of microsatellites have been thoroughly reviewed, and some of the additional models include the two-phase, generalized stepwise (GSMM), and K-allele models (Di Rienzo et al. 1994; Bhargava and Fuentes 2010; Kelkar et al. 2011).

Another difference between the IAM and SMM is that in contrast to the IAM, homoplasy is allowed within the SMM. Ignoring recombination, homoplasy occurs when two individuals with different ancestries at a locus mutate to the same allele and become identical only in state, and not by descent. Homoplasy caused by mutation is expected to occur relatively often for microsatellites compared to other markers because of their allele size constraints and high mutation rates (Kimura and Crow 1964; Estoup et al. 2002). Estoup et al. (2002) showed using simulations that for reasonable mutation rates, a large percentage of alleles are homoplasious under various conditions of heterozygosity, population size, and divergence time. Indeed, high rates of homoplasy have been empirically detected at various levels of incidence in numerous organisms (Lia et al. 2007; Anmarkrud et al. 2008; Barkley et al. 2009; Queloz et al. 2010). Moreover, because microsatellites are almost exclusively genotyped by amplicon length variation, additional causes of homoplasy that would otherwise be detectable by direct sequencing need to be considered (Barthe et al. 2012). First, different microsatellite alleles may be obscured due to insertions or deletions within the flanking region. Homoplasy may also go undetected among individuals with identical amplicon lengths due to hidden variation in the form of point mutations in the microsatellite itself or the flanking region. Collectively, homoplasy is often cited as a significant drawback in the use of microsatellites as genetic markers (Rousset 1996; Estoup et al. 2002; Bhargava and Fuentes 2010; Haasl and Payseur 2011).

There are additional problems with modeling mutational processes. Selecting loci for their high levels of polymorphism in the development phase creates an ascertainment bias that can exacerbate the problems with microsatellites that are associated with high mutation rates (see discussions in Eriksson and Manica 2011; Guillot and Foll 2009; Haasl and Payseur 2011; Li and Kimmel 2013; Petit et al. 2005; Väli et al. 2008). First, the reliability of allele frequency estimation is likely to suffer for highly polymorphic loci. The sample sizes typically employed in population genetic studies (e.g., 30 individuals per population) may (Kalinowski 2005; Hale et al. 2012) or may not (Nei 1978; Ruzzante 1998; Fung and Keenan 2014) be sufficient for accurate estimation of allele frequencies. While a significant potential problem, the uncertainty suggests that the question of sample size might best be verified in empirical studies on a case-by-case basis. Second, loci mutating at a high rate may violate demographic model assumptions, such as mutation–migration–drift equilibrium. Reported mutation rates range from 10^−6^ to 10^−2^ and can vary across loci depending on species, genomic context, repeat size, and nucleotide composition (Ziegler et al. 2009; Bhargava and Fuentes 2010; Grover and Sharma 2011). Moreover, there is ample evidence that mutation rates at a single locus may vary depending on allele length, sex, or taxonomic group (Bhargava and Fuentes 2010; Kelkar et al. 2010; Anmarkrud et al. 2011; Aandahl et al. 2012; Chapuis et al. 2012). Alleles with a higher number of repeats often mutate at a higher rate (Bhargava and Fuentes 2010), and the relationship between length and rate has been reported to be exponential instead of linear (Wierdl et al. 1997; Lai and Sun 2003; Whittaker et al. 2003; Kelkar et al. 2008; Leclercq et al. 2010). Recently proposed models, such as the proportional slippage/point mutation model, allow for heterogeneity in mutation rates within loci (Calabrese et al. 2001). A logistic mutation model was reported to best describe mutation at several human Y-chromosomal microsatellites that show a directional mutation bias (Jochens et al. 2011). However, in this review, we did not identify any methods commonly used to analyze microsatellite data that incorporate these newer, more realistic models.

Microsatellites have an obvious lower bound of zero repeats, and it is hypothesized that a minimum number of repeats is required to facilitate mutation by DNA slippage (Bhargava and Fuentes 2010; but see Kelkar et al. 2010; Leclercq et al. 2010). In addition, microsatellites appear to have an upper limit of allele size. A finite size range can cause an inferential bias that must be accounted for in a model, but the allele size limit of a given microsatellite is difficult to empirically determine (Nielsen and Palsbøll 1999). Typical microsatellite mutation models assume a random walk, or that the gain or loss of a repeat unit(s) is equally likely. However, mutations at some loci have been reported to be biased toward the gain or loss of repeats (Bhargava and Fuentes 2010). Therefore, the fit of a mutation model, the performance of methods when the model is violated, and the possibility of mutation rate varying across and within loci should be considered and evaluated to improve the reliability of inferences in population genetic studies.

### Future outlook for microsatellites

Microsatellites have been the most frequently used genetic marker in population genetics, but the selection of microsatellites over SNPs for a given system may be questionable due to the aforementioned properties of microsatellites, their potential to vary among loci, and uncertainties related to allele frequency estimation. On a per-locus basis, microsatellites retain advantages over SNPs that include higher allelic richness, lower ascertainment bias, and higher analytical power (Schopen et al. 2008; Payseur and Jing 2009; Sun et al. 2009; Guichoux et al. 2011; Haasl and Payseur 2011). Evaluation of these two markers for inferring heterozygosity-fitness correlations have found microsatellites to be inferior to (Väli et al. 2008), similar to (Ozerov et al. 2013; Miller et al. 2014), or better than (Ljungqvist et al. 2010; Forstmeier et al. 2012) SNPs. Inferences for which SNPs have been reported to be superior to microsatellites include inbreeding (Santure et al. 2010), hybrid detection (Väli et al. 2010), and parentage or kinship analyses (Hauser et al. 2011; Ross et al. 2014). For population structure inference, several studies have shown that microsatellites performed generally better than or similar to a low (10s) to moderate (<300) number of SNPs (Herráeza et al. 2005; Coates et al. 2009; Livingstone et al. 2010; Ciani et al. 2013; Granevitze et al. 2014; Ross et al. 2014), whereas ascertainment from a larger pool improved the performance of SNPs (Glover et al. 2010; Gärke et al. 2012; Ozerov et al. 2013). However, many of the studies comparing microsatellites and SNPs have focused on breed or stock identification in intensively studied systems such as salmon (*Oncorhynchus* sp.) using modest but carefully ascertained sets of loci and/or employing loci developed prior to the genomic era. Using high-throughput methods to develop both microsatellite and SNP markers may provide a fair and accurate representation of marker choice for future population genetics studies of nonmodel species.

In addition to their per-locus advantages, due to their high rate of polymorphism microsatellites are often cited as being very useful for studying recent evolutionary events among subpopulations within an individual species or among closely related species (Goldstein and Pollock 1997; Schlötterer 2001; Tsitrone et al. 2001; Ljungqvist et al. 2010; Karl et al. 2012). In empirical studies, microsatellites have performed equally (Morin et al. 2012) or superior (Narum et al. 2008; Hess et al. 2011; Defaveri et al. 2013) to SNPs for revealing fine-scale processes. However, these studies evaluated only a modest number of markers, and it is often stated that the sheer number of SNP loci that can be obtained using high-throughput sequencing is likely to overcome many of the weaknesses of SNPs compared to microsatellites. Haasl and Payseur (2011) thoroughly evaluated the utility of microsatellites and SNPs for addressing several population genetics questions. They found that SNPs generally had greater power to detect population structure compared to microsatellites, as only a few SNP loci were needed to detect structure between populations with moderate divergence times. However, as divergence time decreased (less than two-tenths of effective population size), exponentially more SNP loci were needed whereas increased requirements for microsatellites were modest (Haasl and Payseur 2011). Narum et al. (2008) observed a similar trend under low differentiation (*F*_ST_ < 0.0004) in simulated data for a static number of loci. It is unknown how many unlinked loci are needed to distinguish such recently diverged populations (Haasl and Payseur 2011) and under which ranges of recombination rate (Haasl and Payseur 2011) and genome size attaining these loci will be practical or even possible. These short evolutionary times may translate to appreciable lengths of real time in years for organisms with sufficiently large products of effective population size and generation time (Haasl and Payseur 2011). Therefore, microsatellites may maintain strengths in fields that are focused on short temporal or spatial scales, for applications that require both good resolution and cross-species range (Buschiazzo and Gemmell 2010; Seeb et al. 2011; Dawson et al. 2013), for their reported influence as functional elements (Haasl and Payseur 2013; Sawaya et al. 2013) such as their importance in human diseases (Pearson et al. 2005; Brouwer et al. 2009), in taxa that are slowly evolving or highly clonal, or to study genome evolution (Stolle et al. 2013) or fast evolving genomic regions such as those rich in transposable elements. Preliminary simulation studies would ensure that microsatellites are appropriate for the questions at hand.

A commonly cited weakness of microsatellites is their high development cost and relatively low throughput when compared to SNPs, but the same technologies that have widened the use of SNPs have also benefited microsatellites in the development phase (Arthofer et al. 2011; Churbanov et al. 2012; Duran et al. 2013; Eschbach and Schöning 2013; Fernandez-Silva et al. 2013; Wei et al. 2014). Amplicon sequencing or second-generation sequencing of libraries enriched by target capture can improve the throughput of the genotyping phase for even a modest number of microsatellite loci (Jennings et al. 2011; Bornman et al. 2012; Grover et al. 2012; Highnam et al. 2013; McCormack et al. 2013; Cao et al. 2014). These protocols can be expanded to include other loci of interest for complementary or separate inferences. In addition to improvements in sample throughput, genotyping microsatellites by sequencing provides data that strengthens population genetic inference with microsatellites, by, for instance, unambiguously determining the repeat copy number and detecting imperfect repeats that could identify homoplasy (Barthe et al. 2012; Grover et al. 2012). Microsatellites may be analyzed jointly with adjacent SNPs to make standard inferences (Ramakrishnan and Mountain 2004; Payseur and Cutter 2006; Payseur and Jing 2009; Sorenson and DaCosta 2011) or to address heretofore intractable problems such as complex nonequilibrium scenarios or estimation of mutation rates (Payseur and Cutter 2006). Finally, due to continually expanding read lengths of high-throughput methods, microsatellite data may already or soon be available as a by-product of methods obtaining genomewide SNPs. Indeed, both nucleotide and microsatellite polymorphism data will be available in abundance once complete genomes are available at population scales.

Given their advantages, the use of SNPs is widely expected to dominate the field of population genetics in the immediate future. To compare recent trends in the use of microsatellites and SNPs in the literature, in September 2014 we downloaded 79,956 records from the Web of Science database of articles published since 2004 having a topic of microsatellites and/or SNPs (see Data Availability for search strings and scripts). In agreement with predictions (Guichoux et al. 2011), in our dataset citations of SNPs have as a whole eclipsed microsatellites, increasing from a SNP:microsatellite article ratio of approximately 1:1 in 2010 to 1.17:1 in 2014. These data were examined further to determine whether these trends were consistent among four groups of journals. The titles of journals that have published at least 100 articles in this dataset were identified as having either humans (*n* = 56) or non-humans (*n* = 84) as the primary subject matter. In addition, PLoS One was considered separately due to its very broad subject area and high volume of published articles. All remaining journals (*n* = 4403) constituted the fourth group. Since 2010, SNPs have outnumbered microsatellites at yearly ratios ranging from approximately 1.6:1 to 3.1:1 in the human group and PLoS One, and 1.07:1–1.4:1 in the group of remaining journals (Table 1). In the core group of non-human journals, however, articles citing microsatellites appreciably outnumber those citing SNPs, although the SNP:microsatellite ratios have increased each year from 0.22:1 in 2004 to 0.65:1 in 2014. Because SNPs appear to be experiencing a lag in adoption despite their known advantages and energetic efforts promoting them (e.g., Allendorf et al. 2010; Helyar et al. 2011; Rowe et al. 2011; Seeb et al. 2011; Ferretti et al. 2013; Andrews and Luikart 2014), the use of microsatellites in populations genetics still warrants attention in the literature in the immediate future.

**Table 1:** Trends in number of articles having microsatellites (MS), single-nucleotide polymorphisms (SNP), or both as a topic in the Web of Science database since 2004

	Non-human^1^			Human^2^		
	MS	SNP	Both	MS	SNP	Both
Year	No. of articles with topic (% of total articles in group)					
2004	1114 (5.2)	248 (1.2)	21 (0.1)	516 (2.8)	304 (1.7)	40 (0.2)
2005	1279 (5.8)	360 (1.6)	80 (0.4)	558 (2.8)	445 (2.3)	44 (0.2)
2006	1395 (5.8)	393 (1.6)	33 (0.1)	484 (2.6)	522 (2.8)	47 (0.3)
2007	1464 (5.8)	477 (1.9)	60 (0.2)	443 (2.4)	629 (3.4)	39 (0.2)
2008	1604 (6.3)	526 (2.1)	73 (0.3)	372 (2.0)	778 (4.1)	47 (0.2)
2009	1945 (7.2)	665 (2.5)	71 (0.3)	363 (1.9)	793 (4.2)	59 (0.3)
2010	1613 (5.8)	782 (2.8)	77 (0.3)	284 (1.4)	882 (4.5)	41 (0.2)
2011	1768 (6.0)	882 (3.0)	89 (0.3)	318 (1.6)	896 (4.6)	30 (0.2)
2012	1856 (6.1)	1055 (3.5)	119 (0.4)	280 (1.4)	792 (3.9)	23 (0.1)
2013	1609 (5.4)	1059 (3.5)	123 (0.4)	270 (1.3)	749 (3.7)	22 (0.1)
2014	975 (5.2)	684 (3.7)	99 (0.5)	213 (1.7)	362 (2.9)	21 (0.2)

	PLoS One^3^			Other^4^		
	No. of articles with topic (% of total)			No. of articles		
2004	–	–	–	1309	807	37
2005	–	–	–	1359	951	24
2006	0 (0.0)	1 (0.7)	0 (0.0)	1578	1119	35
2007	12 (1.0)	27 (2.2)	2 (0.2)	1671	1465	57
2008	17 (0.6)	48 (1.8)	0 (0.0)	1873	1715	64
2009	41 (0.9)	81 (1.8)	8 (0.2)	1993	2047	66
2010	47 (0.7)	137 (2.0)	1 (0.0)	2168	2313	68
2011	117 (0.9)	259 (1.9)	13 (0.1)	2233	2580	71
2012	225 (1.0)	444 (1.9)	19 (0.1)	2229	2834	98
2013	277 (0.9)	530 (1.7)	29 (0.1)	2283	2777	77
2014	163 (0.9)	260 (1.5)	19 (0.1)	1241	1735	38

1 A total of 84 journals having a non-human subject matter that have published at least 100 articles since 2004 with MS, SNP, or both as a topic.

2 A total of 56 journals having a human-related keywords in their title (e.g., human, medicine, cancer, forensic, and pharma) that have published at least 100 articles since 2004 with MS, SNP, or both as a topic.

3 PLoS One is presented separately because of its very high article volume (exceeding all 84 non-human journals combined in 2013), its lack of defined subject area, and publication of an appreciable number of population genetics studies.

4 A total of 4403 journals that have published less than 100 articles since 2004 with MS, SNP, or both as a topic.

Specific information regarding the analysis of microsatellite data includes an earlier review (Pearse and Crandall 2004), a basic outline of methods and programs (Kim and Sappington 2013), and an online list of programs for microsatellite data analysis (http://softlinks.amnh.org/microsatellites.html). While microsatellites continue to become more accessible to researchers that may be inexperienced with their use (De Mita and Siol 2012; Karl et al. 2012; Adamack and Gruber 2014) and possess properties that can significantly restrict their inference capabilities, little consolidated information is available on employing tools and interpreting results specifically for analysis of microsatellite marker data in population genetic studies. In light of this lack of information and the unique properties of microsatellites, the objectives of this review are to (1) outline methods that can utilize microsatellites to answer a range of population genetic questions and (2) identify weaknesses in the performance of these methods with microsatellites.

## Population Structure

Genetic structure develops within a species when it departs from panmixia and forms subpopulations among which exchange via dispersal or mating is impeded (Colonna et al. 2009; Waples and Gaggiotti 2006). Defining and identifying subpopulations is of prime interest to numerous biological disciplines for purposes that include conservation, association mapping, study of adaptation, describing habitats and barriers thereof, and detecting migration (Guillot 2008; Fogelqvist et al. 2010; Palsbøll et al. 2010; Haasl and Payseur 2011). However, there is no universal definition of what constitutes or delimits groups of individuals within a species (see Waples and Gaggiotti 2006 for a review and discussion), and indeed, the definition may vary depending on the organisms or questions being investigated. Regardless of the precise definition, elucidating the genetic structure of an organism is a common and convenient way to infer population structure for most taxa. A further discussion on spatially explicit inference of population structure is found in Appendix S1. Spatially explicit methods are recommended to be used in all analyses even if investigating spatial patterns is not an objective because spatial patterns can confound other population genetic inferences (Meirmans 2012). Comparison between nonspatial and spatially explicit methods, such as principal component analysis (PCA) and spatial PCA (sPCA), may serve to rule out the presence of spatial structure.

### Exploratory methods

Clustering and ordination methods are relatively simple yet powerful exploratory methods for analysis of population structure (Appendix S2). The major assumptions, concerns, and implications of these methods for use with microsatellites are presented in Tables 2 and 3, respectively. We find that despite the potential of ordination methods and cluster analysis, their use with microsatellites in population genetic studies has not been investigated or formalized (Odong et al. 2011). The major limitations of clustering and ordination with respect to their use with microsatellites are as follows: (1) scaling allele frequencies; (2) lack of formal methods to incorporate multiallelic markers into PCA; (3) the confounding effect of linkage disequilibrium; and (4) lack of information on the performance of clustering and cluster validation methods, and on interpreting results. Therefore, careful verification through simulations is recommended when using these approaches with microsatellites.

**Table 2:** Major assumptions of and questions relating to exploratory clustering methods and their implications in population genetic studies using microsatellites

Method^1^	Assumption or question	References	Related issues	References
In general	Qualitative (strict) group membership	Xu and Wunsch (2005)	Fuzzy methods allow partial group membership	Xu and Wunsch (2005)
In general	Distance measure is appropriate for data	Felsenstein (2004)	Microsatellite mutation model is difficult to infer and could vary among loci and be costly to incorrectly specify	This paper, introduction
In general	Is clustering method appropriate for sample?	–	Many clustering methods are available but have not been thoroughly tested with microsatellites and/or complex population models	Odong et al. (2011)
In general	Do clustering results accurately depict structure in data or distance matrix?	–	Many methods for cluster validation exist, but are not easily available to population geneticists, have not been evaluated with microsatellites, and are infrequently applied in population genetic studies	Xu and Wunsch (2005), Odong et al. (2011)
UPGMA	Structure is hierarchical	Kalinowski (2009, 2011)	Cannot depict nonhierarchical structure	Kalinowski (2009, 2011)
UPGMA	Constant molecular clock	Felsenstein (2004)	Distorts results when rate of evolution varies among samples	Felsenstein (2004)
NJ	Relaxed molecular clock	Felsenstein (2004)	Allows rate of evolution to vary	Felsenstein (2004)
NJ	–	–	Ties are possible when clustering tips. When individuals are closely related, this can lead to falsely high bootstrap values	Felsenstein (2004)

1 UPGMA, unweighted pair group method with arithmetic mean; NJ, neighbor joining.

**Table 3:** Major assumptions of and questions relating to ordination analyses and their implications in population genetic studies using microsatellites

Method^1^	Assumption or question	References	Related issues	References
In general	How should results from ordination analyses be interpreted?	–	Identifying biologically important structure among results is an open question	Jombart et al. (2009)
In general	Markers are independent sources of ancestry information	Lawson et al. (2012), Baran et al. (2013)	Correlation of markers due to gametic linkage can distort ordination results and impede interpretation	Patterson et al. (2006), Lawson et al. (2012), Baran et al. (2013)
In general	No missing data	–	It may not be clear how missing values for microsatellite loci should be replaced	This paper
In general	Data are noncompositional, relationships between variables are linear	Jombart et al. (2009)	Each allele at a microsatellite locus is treated as a different marker, which creates groups of compositional data	Patterson et al. (2006), Jombart et al. (2008), Odong et al. (2013)
			Microsatellites not formally incorporated into PCA. Analysis combining %PCA with multiple co-inertia analysis may be required	Laloë et al. (2007)
			Little information available on performing transformations to correct for nonlinearity and/or compositional microsatellite data	This paper
PCA	Depicts allele frequency variance, assumes homogeneous variances among alleles	Jombart et al. (2009)	Allele frequencies need to be scaled, but the choice of scaling method for microsatellites may not be obvious or accessible	Jombart et al. (2009), Odong et al. (2013)
PCA	How do outliers influence results from PCA?	–	Outliers are likely to dominate the results and hamper interpretation of other population structure.	Serneels and Verdonck (2008), Zhang et al. (2009)
PCoA	Depicts distance, assumes distance measure is appropriate for data	Jombart et al. (2009)	Microsatellite mutation model is difficult to infer and is costly to incorrectly specify	This paper, introduction
DAPC	Describes between-population variation only	Jombart et al. (2010)	Depends on assumptions of PCA and chosen clustering method	Jombart et al. (2010)

1 PCA, principal component analysis; PCoA, principal coordinate analysis; DAPC, discriminant analysis of principal components.

Because PCA formulates principal axes to maximize variance, scaling the data should be carefully considered to account for bias induced by heterogeneous variances among data points (Jombart et al. 2009). It has been proposed that the variance be standardized by the square root of the product of allele frequencies (Jombart et al. 2009) or, specifically for microsatellites, by the standard deviation after centering each allele on zero (Odong et al. 2013). The latter scaling method was reported to greatly improve accuracy of downstream population assignment methods (Odong et al. 2013). Scaling allele frequencies is highly recommended for any marker (Jombart et al. 2009), but to our knowledge, only Odong et al. (2013) have proposed and evaluated scaling a microsatellite dataset. Custom scaling may require direct manipulation of allele frequency matrices (Odong et al. 2013), which is an unfamiliar task for many users and may be considered inappropriate if performed after the data have been collected.

In addition to scaling, the multiallelic nature of microsatellites has not been formally incorporated into ordination (Limpiti et al. 2011), and it is not always clear how software packages handle multiallelic data. Each allele is treated as a different marker in *adegenet* and in several instances in which this problem is explicitly mentioned (Limpiti et al. 2011; Liu and Zhao 2006; Odong et al. 2013; Patterson et al. 2006). This artificial expansion of the dataset creates sets of compositional measurements and thus dependence among these alleles, because their allele frequencies have a constant sum (Jombart et al. 2009). Distinct from scaling, transformation may be required to correct for problems associated with compositional data and/or problems due to nonlinear structure (Jombart et al. 2009). It was suggested in Patterson et al. (2006) to analyze microsatellites directly as continuous variables, as is done for most data in other fields, and perform PCA after normalization. However, use of raw allele sizes or repeat number instead of allele frequencies could make this analysis distance-like, and it is unclear whether it is even possible to incorporate microsatellites into PCA.

To work around these obstacles, it may be necessary to use the method performed by (Laloë et al. 2007) that uses %PCA (de Crespin de Billy et al. 2000), which is designed for compositional data, and multiple co-inertia analysis (MCOA), which is among a class of ordination methods that are powerful for their ability to associate different types of data or results from different analysis methods (Jombart et al. 2009). In this method, each locus is analyzed separately using %PCA, and then, MCOA is to summarize signals of structure common among the microsatellite loci and detect discordant signals (Laloë et al. 2007). Although not highlighted, an example of this approach is provided in the documentation for *adegenet* (Jombart 2008). While MCOA requires data to be in the form of allele frequencies per subpopulation because it evaluates the congruence of structure already inferred, this allows the flexibility to define different patterns of population structure and to evaluate the support for these patterns from each marker.

A widely cited benefit of population genetic analysis with ordination methods is that they do not assume gametic linkage equilibrium (Jombart et al. 2009). However, correlation of markers due to gametic linkage can alter results of population structure inference (Patterson et al. 2006; Lawson et al. 2012; Baran et al. 2013). Lawson et al. (2012) demonstrated that linkage disequilibrium can obscure structure in a large SNP dataset, and developed a modified form of PCA incorporating linkage information that is able to detect more fine-scale structure. The ad hoc method proposed by Patterson et al. (2006) could be used in microsatellite datasets if linkage is suspected or known by conducting analyses with one in the pairs of linked loci removed.

Cluster validation methods are important for evaluating the fit of the cluster output to the distance matrix, determining the number of clusters, and choosing which clustering method best describes the data (Odong et al. 2011). Few methods (e.g., Kalinowski 2009) are available to validate results by hierarchical clustering (reviewed and evaluated by Odong et al. 2011). While the use of hierarchical clustering with microsatellites has received little formal attention, the two most widely used methods (unweighted pair group method with arithmetic mean [UPGMA] and Ward) have been shown to perform well (Odong et al. 2011). Given that rates of evolution may vary among microsatellite loci, the choice between a clustering method with a strict (UPGMA) or relaxed (neighbor joining, NJ) molecular clock may have implications when inferring phylogenies. In addition, Kalinowski (2009) showed that NJ performed well at depicting relationships for both a bifurcating fragmentation and linear stepping stone model, whereas UPGMA accurately depicted only the fragmentation population model. Interpreting results from an ordination analysis in terms of assigning individuals to clusters and identifying and quantifying population differentiation is often not straightforward (Reich et al. 2008). While performing clustering on PCA results does not properly assign individuals to groups according to genetic distances between subpopulations (Intarapanich et al. 2009), Odong et al. (2013) showed that performing hierarchical clustering on scaled PCA analysis of a microsatellite dataset significantly improved assignment of accessions of coconut (*Cocos nucifera* L.) to their original group. Discriminant analysis of principal components describes between-subpopulation variation only and is a powerful tool for population genetic inference using microsatellites that can outperform the STRUCTURE program when inferring the number of subpopulations (*K*) (Jombart et al. 2010).

### Descriptive statistics

Descriptive statistics as discussed in this review include measures such as fixation statistics and diversity-based statistics. Brief background on these statistics is given in Appendix S3. In brief, several properties of these statistics that are relevant to their use with microsatellites include *F*_ST_'s original derivation for biallelic data (Meirmans and Hedrick 2011), the representation of SMM-based parameters of evolutionary distance in addition to differentiation (Holsinger and Weir 2009), the high sampling variance of parameters that include allele size (Slatkin 1995; Gaggiotti et al. 1999; Balloux and Goudet 2002), and the advantages of entropy-based methods (Sherwin et al. 2006; Sherwin 2010; Andrew et al. 2012; Blum et al. 2012).

In contrast to clustering and ordination, the properties and performance of descriptive statistics have been and are under active investigation. While this research has identified several well-known issues with respect to microsatellites, it has not always added clarity to how these markers should be applied and interpreted for any marker type. The major assumptions of descriptive statistics and their implications with microsatellites are summarized in Table 4. Here, we discuss the following main challenges using descriptive statistics with microsatellites: (1) depression of *F*_ST_ at high mutation rates; (2) making comparisons when groups have large allele size differences; (3) sensitivity of *R*_ST_ to deviations from the SMM; (4) choosing the more accurate measure between *F*_ST_ or *R*_ST_; (5) homoplasy and null alleles; (6) confusion between parameters and estimators, and the identity of various statistics; and (7) the confusing debate surrounding these statistics regarding microsatellites in particular and population genetics in general.

**Table 4:** Major assumptions of and questions relating to descriptive statistics and their implications in population genetic studies using microsatellites

Method	Assumption or question	References	Related issues	References
In general	Diploid genome	Dufresne et al. (2014)	For haploids or especially polyploids, options (in terms of both statistics and packages) are fewer	Dufresne et al. (2014)
*F*-statistics	Biallelic markers^1^ under infinite allele model of mutation. Often estimated using heterozygosity	Holsinger and Weir (2009), Meirmans and Hedrick (2011)	*F*_ST_ and relatives depressed under high diversity, requiring adjusted versions, especially problematic when mutation rate exceeds migration rate	Hedrick (2005), Jakobsson et al. (2013)
			Requires use of unbiased estimators of heterozygosity. Cannot compare subpopulations or loci with different levels of gene diversity	Meirmans and Hedrick (2011)
*F*-statistics	Infinite island model of population structure	Meirmans and Hedrick (2011)	Violation of migration assumptions require *F*_ST_ to be estimated pairwise or using alternatives (extension of *θ*, or *F*-model)	Weir and Hill (2002), Gaggiotti and Foll (2010)
			Correlation of allele frequencies among populations can cause overestimation using most methods	Fu et al. (2005)
*R*_ST_, *ρ*_ST_ fire	Stepwise mutation model (SMM)	Chakraborty and Nei (1982), Slatkin (1995), Rousset (1996)	Likely confounded by deviations from SMM. Levels of diversity and structure in the sample likely influence relative performance of *F*_ST_ and *R*_ST_	Balloux et al. (2000), Balloux and Goudet (2002), Balloux and Lugon-Moulin (2002)
			Parameters including allele size are associated with high variance; should be estimated using analysis of molecular variance (AMOVA)	Michalakis and Excoffier (1996), Balloux and Goudet (2002)
*D*	Depends only on allelic differentiation	Jost (2008)	Can be sensitive to markers with high mutation rates	Leng and Zhang (2011, 2013)
In general	Which statistics should be employed?	–	Recommended to report as many as possible with microsatellites and ensure clarity in distinguishing parameters and estimators, such as for *R*_ST_, *ρ*_ST_, or Φ_ST_	Heller and Siegismund (2009)
In general	How should *F*-statistics be interpreted?	–	For any parameter: Microsatellites may substantially underestimate population structure, and interpretation has been described as “dangerous.”	Balloux and Lugon-Moulin (2002), Leng and Zhang (2011)

*G*_ST_ and some implementations of *θ* can be used for multiallelic markers.

As a measure of fixation derived from population genetic theory, *F*_ST_ (Wright 1943) not only describes the current state of population structure, but it is influenced by past evolutionary processes such as mutation (Holsinger and Weir 2009; Meirmans and Hedrick 2011). Use of fixation indices with microsatellites can be problematic because they are depressed at high mutation rates (Balloux et al. 2000; Hedrick 1999, 2005; Meirmans 2006; Jost 2008; Kronholm et al. 2010; Song et al. 2011; Whitlock 2011; Wang 2012). This is of particular concern when the mutation rate is similar to or exceeds the rate of migration (Balloux and Lugon-Moulin 2002; Meirmans and Hedrick 2011; Whitlock 2011), but not under nonequilibrium conditions (Leng and Zhang 2013). For example, *G*_ST_ (Nei 1973) has been shown to approach zero in some cases when differentiation between subpopulations is complete (Carreras-Carbonell et al. 2006). *F*’_ST_ and *G*’_ST_ are parameters standardized for within-subpopulation diversity to account for cases when the parameters are small despite subpopulations sharing few alleles (Hedrick 1999, 2005; Meirmans and Hedrick 2011). In addition, due to their dependence on diversity, these statistics cannot be used to reliably compare loci or subpopulations with different levels of gene diversity (Charlesworth 1998; Hedrick 1999; Jost 2008; Meirmans and Hedrick 2011; Jakobsson et al. 2013) unless methods such as rarefaction are used (Eriksson and Manica 2011). Except for the SMM-based parameter *R*_ST_ (Chakraborty and Nei 1982; Slatkin 1995) (Appendix S3), unbiased estimators of heterozygosity should be used for estimating *F*-statistics, and extra caution should be exercised when making comparisons of subpopulations with different gene diversity levels (Beaumont and Nichols 1996; Leng and Zhang 2011; Meirmans and Hedrick 2011).

Because longer microsatellite alleles mutate at a higher rate, comparisons among subpopulations or species with appreciable size differences between alleles may be biased. Correlation of mean number of repeats with various diversity measurements has been reported in humans (Pemberton et al. 2009), select studies in *Drosophila melonagaster* (Colson and Goldstein 1999; Bachtrog et al. 2000), and between conifers and angiosperms (Petit et al. 2005). Procedures such as standardization of diversity measures to mean number of repeats are recommended to avoid the significant diversity artifact created by microsatellite length differences (Petit et al. 2005). A natural extension of this recommendation is to analyze all data using the number of repeats instead of allele sizes, which is incorporated into several microsatellite-specific distance measurements, but it is unclear how this practice would influence exploratory analyses such as PCA.

Although not influenced by mutation rate or within-subpopulation gene diversity, *R*_ST_ is widely reported to be quite sensitive to deviations from the stepwise mutation model (Balloux et al. 2000; Holsinger and Weir 2009; Meirmans and Hedrick 2011; Whitlock 2011), but not under drift–mutation–migration equilibrium when mutation follows the two-phase mutation model instead of the SMM (Song et al. 2011). *R*_ST_ may perform better at describing population structure than *F*_ST_ when diversity is high (>70%) or when structure is strong because *R*_ST_ ignores the contribution of the stepwise mutation process to differentiation when inferring migration (Balloux and Goudet 2002; Balloux and Lugon-Moulin 2002). However, even when mutation closely follows the SMM, due to its high variance *R*_ST_ may be outperformed by *F*_ST_ when diversity is low (<50%) or there is weak structure (Balloux and Goudet 2002; Balloux and Lugon-Moulin 2002).

Despite the difficulty in directly determining the mutation model of a given microsatellite, it is possible to determine whether the mutation model or rate is confounding parameter estimation in population studies. The allele size permutation test, available in the program SPAGeDi (Hardy and Vekemans 2002), estimates if stepwise mutations have added to differentiation and, therefore, if *R*_ST_ is more appropriate to infer population structure or migration compared to a biased *F*_ST_ (Hardy et al. 2003). In contrast, a nonsignificant test suggests that mutation is unimportant relative to drift, and thus, that *F*_ST_ is preferable over *R*_ST_ (Hardy et al. 2003). If the SMM can be assumed, then the permutation test can also compare the influence of mutation rates with migration rates or with divergence times (Hardy et al. 2003). However, this test is blind to other confounding influences, such as model violations for *F*_ST_ or variance for *R*_ST_, and is recommended to be applied only to loci with five or more alleles (Hardy et al. 2003). As implemented in the program BOTTLENECK (Piry et al. 1999), the heterozygosity-excess test for detecting changes in population size (discussed in a later section) can be used to infer if a microsatellite locus is evolving according to the IAM, two-phase model, or SMM, if it assumed the locus is at mutation–drift equilibrium (Cornuet and Luikart 1996). Comparison of *G*_ST_ and the diversity-based parameter *D* (Jost 2008) (Appendix S3) could also inform on mutation rates and divergence times (Leng and Zhang 2013). Tests have been used in empirical studies to detect violations of the SMM (Di Rienzo et al. 1994; Nielsen and Palsbøll 1999), but to our knowledge, these tests are not available in computer programs. Entropy-based measures may also be used to infer the mutation model of microsatellite loci. To do so, Sherwin et al. (2006) first estimated Θ using both heterozygosity and the entropy parameter ^*S*^*H* (Shannon 1948a,b), each assuming either the IAM or the SMM. The model that has the smallest relative difference between the fixation and entropy-based theta estimates is proposed as evidence for that mutation model operating at that locus (Sherwin et al. 2006).

Homoplasy caused by mutation may influence inferences using microsatellites because it depresses gene diversity and the level of allelic differentiation, which may lead to underestimation of population differentiation (Estoup et al. 2002; Sefc et al. 2007). Homoplasy is a particular concern for analysis of populations with large effective population sizes, or loci with high mutation rates or strict allele size constraints (Nauta and Weissing 1996; Estoup et al. 2002). The influence of homoplasy on differentiation estimates is reduced when migration is high or when subpopulations recently diverged (Rousset 1996; Estoup et al. 2002). While homoplasy should be taken into account in certain conditions, it is likely of minor concern in most population genetic studies (Estoup et al. 2002).

In addition to homoplasy, mutations in the region flanking a microsatellite can cause null alleles, or alleles that fail to amplify. Null alleles may lead to an overestimation of population differentiation because they reduce gene diversity (Chapuis and Estoup 2007). The occurrence of null alleles, or homozygote excess, may be estimated upon initial data analysis by one of several methods (e.g., Chapuis and Estoup 2007; Chapuis et al. 2008; Van Oosterhout et al. 2004; Wang et al. 2012), but their bias is only infrequently corrected for when determining population differentiation (Chapuis and Estoup 2007). While frequencies of null alleles up to 8% may cause only minimal bias in estimation of some population genetic parameters (Oddou-Muratorio et al. 2009), correction may not sufficiently reduce bias for inferring population structure and may actually exacerbate it (Chapuis and Estoup 2007). Dąbrowski et al. (2014) reported poor agreement among five methods of estimating null alleles in nonequilibrium conditions, although biases of these methods with respect to both false negatives and positives could be useful for null allele inference. However, the method developed by Wang (2012), which simultaneously estimates null alleles and corrected inbreeding coefficients and heterozygosity, was not evaluated. While it is important to consider null alleles in the analysis of any microsatellite dataset, Dharmarajan et al. (2013) proposed that sampling and locus-specific effects could create artifacts in calculations of heterozygosity that lead to an overestimation of null alleles.

There is some confusion in the literature regarding the identity and use of SMM-based parameters and estimators, primarily over the relationship of *R*_ST_ and another SMM-based parameter, *ρ*_ST_ (Rousset 1996) (Appendix S3). Holsinger and Weir (2009) state that *R*_ST_ and Φ_ST_ are specific to microsatellite and haplotype data, respectively, but *R*_ST_ is a theoretical parameter and Φ_ST_ is an estimator that can be applied to either haplotypes (Excoffier et al. 1992) or microsatellites (Michalakis and Excoffier 1996). In empirical studies, calculation of *R*_ST_ in the Materials and Methods section is accompanied by concurrent citations of both Slatkin (1995) and Rousset (1996). However, Michalakis and Excoffier (1996) clearly identify the two parameters as distinct: “The equilibrium value of the parameter (*ρ*_ST_) estimated by Φ_ST_ for microsatellite data has been determined by Rousset (personal communication), who also first derived the relationship between *R*_ST_ and Φ_ST_.” This distinction is also clearly conveyed by Rousset (1996) in the section entitled “Estimation and relationship to Slatkin's *R*_ST_.” Care should be used when calculating and reporting these estimators of SMM-based parameters.

Due to the considerable debate regarding their use in population genetics (Appendix S3) and the context-dependent of some aspects of their performance (e.g., mutation model), descriptive statistics should be applied judiciously and validated through simulations. Microsatellites may lead to substantial underestimates of population structure when migration is low, regardless of the parameter used to estimate it (Balloux et al. 2000). Even excluding the problematic properties of microsatellites, interpretation of *D*, *F*_ST_, *G*_ST_, and *R*_ST_ have all been described as “dangerous” (Balloux and Lugon-Moulin 2002; Leng and Zhang 2011). All parameters, such as the *F*_ST_-estimator *θ* (Weir and Cockerham 1984; Weir and Hill 2002), *G*_ST_, *G’*_ST_, *R*_ST_, and *D* should be reported to facilitate additional interpretation and meta-analysis (Heller and Siegismund 2009). However, due to their properties, indices such as *F*_ST_, *R*_ST_, and *D* can be useful for complementary analyses (Balloux and Lugon-Moulin 2002; Segarra-Moragues and Catalán 2008; Song et al. 2011; Whitlock 2011; Leng and Zhang 2013). The confidence interval or standard error of parameter estimates should be determined using bootstrapping or jackknifing approaches to allow an assessment of the parameter's reliability (Gerlach et al. 2010; Meirmans and Hedrick 2011).

### Model-based clustering

Parametric methods that implement population genetic assumptions are powerful and popular tools for inference of population genetic structure. They are particularly useful for relating observable structure to genetic structure, and for detecting cryptic structure, or structure that is only apparent genetically (Pritchard et al. 2000). These methods address central questions such as the detection of structure, estimation of the number of subpopulations, and the assignment of individuals to these subpopulations. Commonly used model-based methods for inferring population structure include STRUCTURE (Pritchard et al. 2000) and Bayesian analysis of population structure (BAPS) (Corander et al. 2003). Details on the models and algorithms employed in these programs are found in Appendix S4, and an overview of methods for model-based estimation of the number of subpopulations is given in Appendix S5. The central assumptions of these methods and the respective problems with their use with microsatellites are summarized in Table 5. Because these methods are stochastic, results from individual runs can vary and lead to irreproducible results in an appreciable number of cases (Gilbert et al. 2012). Vigilance is required to ensure that an appropriate number of steps and replicate runs have been performed to achieve the best possible accuracy and precision (Gilbert et al. 2012). Scripting programs are available to aid users at setting up the many required runs (Chhatre 2012; Besnier and Glover 2013).

**Table 5:** Major assumptions of and questions relating to model-based clustering and their implications in population genetic studies using microsatellites

Method	Assumption or question	References	Related issues	References
In general	Individual runs are stochastic and may settle on local optima	Gilbert et al. (2012)	Ensure that a sufficient number of steps and runs have been performed	Gilbert et al. (2012)
BAPS and STRUCTURE	Hardy–Weinberg equilibrium within populations	Pritchard et al. (2000), Corander et al. (2004)	No inbreeding; if suspected, use InStruct	Gao et al. (2007)
			Individuals are not related by direct descent; related individuals should be removed prior to analysis	Anderson and Dunham (2008), Rodríguez-Ramilo and Wang (2012)
BAPS	Gametic linkage equilibrium within populations. Tight (BAPS) or loose (STRUCTURE) linkage allowed for one model only	Falush et al. (2003), Corander and Tang (2007)	Use of linkage model requires data be haploid, or phased data from a diploid or tetraploid	Corander and Tang (2007)
STRUCTURE			Sufficient number of markers should be unlinked. Phasing optional for diploids, required for polyploids	Falush et al. (2003), Kaeuffer et al. (2007)
BAPS and STRUCTURE	Is the information content of the dataset sufficiently high?	–	Population structure: Incomplete lineage sorting may confound inference when using as many 50 microsatellite loci	Orozco-terWengel et al. (2011)
			Admixture: If few microsatellite loci are used, the sample should include a significant number of pure individuals	Pritchard et al. (2000), Vaughan et al. (2009)
STRUCTURE and NewHybrids			Hybrid identification: reliable only for many loci (>24–50), especially when differentiation is low	Vähä and Primmer (2006), Fitzpatrick (2012)
STRUCTURE	Recessive allele model: null allelesare due to polymorphism	Falush et al. (2007)	Not appropriate for data that are missing due to experimental error	Falush et al. (2007)
STRUCTURE	Prior population^1^ and correlated allele frequency models allow detection of weak structure	Falush et al. (2003), Hubisz et al. (2009)	Using standardized values, performance of STRUCTURE and BAPS declines at standardized *F*_ST_ values of 0.28. 97% accuracy was attained only at *F*_ST_ = 0.39	Latch et al. (2006)
BAPS	Two models that incorporate population information a priori are available	Corander et al. (2006, 2003)		

BAPS, Bayesian analysis of population structure.

1 The two prior population models, LOCPRIOR and USEPOPINFO, should be used for weak and strong structure, respectively.

Here, our literature review highlights questions on the power of model-based methods to make inferences in situations to which they are commonly applied. The major problems of use of model-based methods with microsatellites are as follows: (1) inference of weak structure; (2) the confounding effect of incomplete lineage sorting; (3) the need for a large number of loci (>50) to accurately identify population structure, admixture, or hybrids; and (4) null alleles. To ensure the study design allows the objectives to be reliably addressed, researchers are strongly encouraged to perform power analysis by analyzing simulated datasets (Orozco-terWengel et al. 2011; Vähä and Primmer 2006).

#### Weak structure

Inferring population structure when differentiation between subpopulations is weak often is of interest to researchers, but it is a difficult analytical problem. Latch et al. (2006) evaluated BAPS v3.1 (“cluster groups of individuals” option) and STRUCTURE v2.1 (Falush et al. 2003) under conditions of low population differentiation using simulated loci similar to microsatellites and found that even the earlier STRUCTURE models perform well at low levels of genetic differentiation (0.02 < *F*_ST_ < 0.10), but fails at lower values (Duchesne and Turgeon 2012). When differentiation values were standardized for heterozygosity, however, the performance of both BAPS and STRUCTURE declined at standardized *F*_ST_ values as high as 0.28, and a value of 0.39 was needed for individual assignment accuracy to reach 97% (Latch et al. 2006). FLOCK has recently been extended to microsatellites and was reported to be more accurate compared to STRUCTURE at low levels of differentiation (*F*_ST_ ≤ 0.05) (Duchesne and Turgeon 2012).

Nongenetic information such as phenotypes or sampling group may be informative to population structure inference, especially when genetic structure is weak. Both STRUCTURE and BAPS possess methods to incorporate this a priori information (Appendix S4). To provide an objective evaluation of the association of user-defined prior information with detected structure, Gayevskiy et al. (2014) developed OBSTRUCT, which uses correlation and the multivariate method canonical discriminant analysis to postprocesses results from any method which infers ancestry proportions, especially STRUCTURE, BAPS, or InStruct. Although validated with several simulated and empirical microsatellite datasets, Gayevskiy et al. (2014) did not evaluate either of STRUCTURE's prior population models (Hubisz et al. 2009) nor incorporate them into OBSTRUCT's workflow.

The high differentiation threshold found by Latch et al. (2006) may be due to their use of only 10 loci in their simulations (Colonna et al. 2009). In contrast, Colonna et al. (2009) compared reconstructed family histories in two Italian villages to data from a panel of 1122 microsatellites. They found that 239 of these loci were sufficient for STRUCTURE to accurately identify the population structure for an extremely low value of *F*_ST_ = 0.008 (Colonna et al. 2009). Thus, the information content of a dataset likely has a significant influence on the performance of STRUCTURE.

#### Dataset information content

Orozco-terWengel et al. (2011) used BAPS to analyze numerous subsets of 137 microsatellites in *Drosophila melanogaster* and found that different subsets yielded different conclusions about population structure despite all receiving high statistical support. Because similar results were also obtained in cursory evaluations with STRUCTURE, Orozco-terWengel et al. (2011) concluded that incomplete lineage sorting could confound structure inference with the number of microsatellite loci typically employed in population genetic studies, particularly for weak population differentiation and regardless of the algorithm employed. Moreover, they found that statistically significant population structure can still be detected with an insufficient number of loci, and recommend simulations be used for a power analysis of the number of loci needed for reasonably accurate inferences (Orozco-terWengel et al. 2011). FLOCK, for example, returns an “undecided” result when the data are not informative enough for population structure inference (Duchesne and Turgeon 2012).

The problem of too few loci can be particularly troublesome when inferring admixture. Vaughan et al. (2009) examined an experimental cross of mice and found that 11 microsatellite loci were generally insufficient for correctly inferring admixture when individuals from the founding subpopulations were not included in the analysis. When a large proportion of individuals in the dataset are admixed, Pritchard et al. (2000) observed that estimates of ancestry coefficients may only be reliable for a large number of loci. Plotting the distribution of ancestry coefficients for each individual can help assess the confidence of these estimates (Anderson and Dunham 2008). The admixture models implemented in STRUCTURE are designed for detecting admixture at any time point and allow for the ancestral subpopulations to be unsampled. However, very recent admixture events may be detected from the sampling of hybrid individuals, and detecting these events may be a central goal of studies when subpopulations come into contact via dispersal or from sharing a border (Anderson and Thompson 2002; Vähä and Primmer 2006). When parental subpopulations are well characterized and the sample is known to contain pure and hybrid individuals, hybrid-specific detection methods are likely to outperform model-based clustering methods (Anderson and Thompson 2002; Vähä and Primmer 2006; Sanz et al. 2009).

One hybrid-specific method, NewHybrids, analyzes genotype frequencies to assign individuals to genotype frequency classes consisting of pure, backcrossed, or hybrid (*F*_1_ or *F*_2_) (Anderson and Thompson 2002). NewHybrids analyzes data using Bayesian methods similar to STRUCTURE. Vähä and Primmer (2006) used simulated data to show that STRUCTURE and NewHybrids perform similarly at hybrid identification, and the advantage conferred by including reference information was not large. STRUCTURE was shown to readily identify hybrid individuals even when subpopulations are only weakly differentiated, whereas NewHybrids performed poorly compared to STRUCTURE at the lowest level of differentiation (*F*_ST_ = 0.03) (Vähä and Primmer 2006). However, similar to the related question of admixture, a large number of microsatellite loci (≥24) were required for accurate hybrid identification at low levels of differentiation (Vähä and Primmer 2006). Although both methods performed similarly overall, only NewHybrids efficiently distinguished among the genotype frequency classes, but only for 48 loci and when differentiation was high (*F*_ST_ = 0.21). Similarly, Fitzpatrick (2012) concluded that at least 50 ancestry-informative loci are needed to allow accurate identification of hybrids. Additionally, as mentioned above for admixture, STRUCTURE can overestimate the amount of admixture and result in the misclassification of nonhybrid individuals as hybrid (Bohling et al. 2013). Comprehensive reviews, evaluations, and comparisons of these methods are available (Anderson and Thompson 2002; Sanz et al. 2009; Väli et al. 2010; Verdu and Rosenberg 2011; Twyford and Ennos 2012; Uwimana et al. 2012). Collectively, admixture results should be interpreted with caution, especially when too few loci are used or when an insufficient number of individuals from the ancestral parent subpopulations are included or detected in the analysis (Pritchard et al. 2010). In addition, performing simulations is recommended to assess the confidence level of inferences made regarding hybridization in a population (Vähä and Primmer 2006).

#### Population models

Although most model-based clustering methods were not derived from a particular model of population structure, the true model could have a confounding influence on making accurate inferences (Choi and Hey 2011). For instance, clear Hardy–Weinberg subpopulations are often not distinguishable from the data (Kalinowski 2011; Schwartz and McKelvey 2008) when trying to delimit upper levels of hierarchical structure, which may confound model-based clustering programs (Rodríguez-Ramilo and Wang 2012). Jombart et al. (2010) showed that STRUCTURE is highly effective at assigning individuals for both the island and hierarchical models of population structure. However, STRUCTURE did not estimate the correct number of clusters under the hierarchical model and failed at both tasks for data simulated under two different stepping stone models (Jombart et al. 2010). In contrast to Jombart et al. (2010), Kalinowski (2011) performed coalescent simulations of microsatellites under a simple hierarchical fragmentation model and showed that STRUCTURE could not assign individuals to the correct group of subpopulations. Kalinowski (2011) concluded that results from STRUCTURE are not appropriate for describing differentiation or relatedness among clusters and that a simple neighbor-joining tree derived from an unbiased distance measure is more effective at describing these relationships. Unlike STRUCTURE, BAPS accounts for clustering at multiple levels and is expected to perform well for datasets with hierarchical structure (Corander et al. 2004), but the performance of BAPS under various demographic models has not been extensively evaluated to our knowledge. Results should therefore be evaluated and carefully interpreted if the organism under study is suspected to evolve under a complex population model.

Isolation by distance is a type of population structure in which genetic similarity is inversely related to geographical distance due to the organism's limited dispersal ability (Meirmans 2012; but see Puebla et al. 2012). Because isolation by distance can be confused with hierarchical structure and vice versa depending on which model is assumed in a given analysis, caution should be used when drawing conclusions about the two models (Meirmans 2012). Procedures for making accurate inferences when isolation by distance or hierarchical structure might be present have been reviewed by Meirmans (2012). A significant problem is that when isolation by distance is present, model-based methods typically detect spurious clusters (Schwartz and McKelvey 2008; Frantz et al. 2009; Safner et al. 2011) and also erroneously identify the borders between subpopulations (Blair et al. 2012). Additional problems of estimating isolation by distance under nonideal conditions such as unequal and changing population sizes have been investigated by Björklund et al. (2010). To increase reliability in border identification, Blair et al. (2012) recommend fixing *K* to the number of subpopulations believed to flank the border.

#### Null alleles

The recessive allele model in STRUCTURE can be useful for studies using microsatellites. Null alleles may bias parametric population structure inference because their presence increases the number of homozygous individuals relative to Hardy–Weinberg equilibrium (Carlsson 2008). However, Carlsson (2008) showed that the influence of null alleles on accurately assigning individuals to subpopulations is only slight. It was concluded that the influence of null alleles is marginal compared to other factors such as the number of loci and strength of population differentiation (Carlsson 2008). Similarly, Dharmarajan et al. (2013) reported that the degree of the Wahlund effect may vary widely among loci and that excesses in homozygosity that are observed at only a few loci are often misinterpreted as being caused by null alleles. However, Dharmarajan et al. (2013) only used summary and *F*-statistics in their analyses and not STRUCTURE. While Carlsson (2008) evaluated the recessive allele model introduced to STRUCTURE by Falush et al. (2007), it remains unclear if methods to correct for null alleles prior to analysis (e.g., Van Oosterhout et al. 2004; Chapuis and Estoup 2007; Wang et al. 2012) are appropriate for model-based population structure inference.

## Migration

In population genetics, migration refers to the dispersal of individuals to geographically separate subpopulations and the subsequent persistence of these individuals within the new subpopulation (Lowe and Allendorf 2010). Migration is a common mechanism that erodes population structure and reduces genetic differentiation between subpopulations; lack of migration is a common mechanism that increases differentiation. Indeed, Wright (1951) posited that only one migrant per generation is sufficient to break population structure (but see Lowe and Allendorf 2010; Waples and Gaggiotti 2006 for discussion on alternative interpretations). Due to the close relationship between population structure and migration, some of the methods used to infer the former are similar to or the same as those used to infer the latter. Methods of migration inference have been reviewed (Manel et al. 2005; Broquet and Petit 2009; Lowe and Allendorf 2010). Briefly, methods that analyze migration are either indirect, which infer migration from genetic signatures among subpopulations, or direct, which identify migrant individuals by their genotype (Broquet et al. 2009; Lowe and Allendorf 2010). Indirect methods perform well when migration is high and are usually meant to infer effective migration, or migration that becomes integrated into the local subpopulation (Broquet et al. 2009). In contrast, direct methods perform well when migration is low (and therefore population structure is strong) and usually infer recent migration by identifying the actual migrant individuals (Waples and Gaggiotti 2006; Lowe and Allendorf 2010). Similar to population structure, there is active debate on the use of descriptive statistics for inferring migration (Appendix S6).

Individual-based clustering methods are direct methods of migration inference that can detect recent migration to a given subpopulation by identifying individual(s) that belong to another, genetically distinct subpopulation. These assignment methods have been thoroughly reviewed by Manel et al. (2005) and include many of the model-based clustering methods discussed above for population structure. The accuracy and efficiency of these methods for detecting migrants is likely closely related to their ability to detect individuals that do not belong with their subpopulation of origin or for which the subpopulation of origin has little or no representation in the dataset. These methods have been shown to perform well at detecting individuals that have migrated, with TESS generally performing better than GENELAND, GENECLUST, and STRUCTURE (Chen et al. 2007).

These assignment methods can perform well at identifying migrant individuals, but they can be outperformed by assignment-based techniques that also infer rates of migration between subpopulations (Waples and Gaggiotti 2006). These methods aim to detect the percentage of migrants in a subpopulation one to a few generations after it occurred (Fraser et al. 2007; Kane and King 2009), and their assumptions (Table 6) differ from the model-based clustering methods above. Wilson and Rannala (2003) developed BayesAss, an assignment method that estimates migration rates and probabilities of ancestry of migrant individuals (Wilson and Rannala 2003). BayesAss detects disruptions to gametic linkage disequilibrium by estimating inbreeding coefficients for each subpopulation and does not depend on Hardy–Weinberg equilibrium (Wilson and Rannala 2003). Like other direct methods, BayesAss performs better at higher levels of differentiation (Wilson and Rannala 2003). Following its limited validation using biallelic markers (Wilson and Rannala 2003), Faubet et al. (2007) performed an extensive evaluation of BayesAss using multiallelic markers and at various levels of migration and population differentiation. BayesAss was found to perform well at migration rates up to 0.1 when model assumptions were met, but violation of assumptions led to a decline in performance when migration rates were greater than 0.01 (Faubet et al. 2007). In addition, the individual migrant probabilities were found to be less than reliable (Faubet et al. 2007). In another study combining simulations with an experimental population in vitro, Mardulyn et al. (2008) found that BayesAss consistently overestimated migration rates. Faubet et al. (2007) and Meirmans (2014) found using simulated datasets and an empirical literature review that the algorithm in BayesAss has an unsatisfactory convergence behavior and provide recommendations for alleviating this condition.

**Table 6:** Major assumptions of and questions relating to inference of migration or population size, and their implications in population genetic studies using microsatellites

Method	Assumption or question	References	Related issues	References
Migration				
BayesAss	Gametic linkage equilibrium within populations	Wilson and Rannala (2003)	Detects shifts in gametic linkage equilibrium to estimate recent migration	Wilson and Rannala (2003)
BayesAss	Low migration rate; immigrants comprise less than one-third of population	Faubet et al. (2007)	When assumptions are violated, inferences using microsatellites may be accurate only for low migration rates (<0.01) and high differentiation (*F*_ST_ > 0.1)	Faubet et al. (2007)
BayesAss	Migration and drift are constant during past few generations	Faubet et al. (2007)		
GENECLASS2	Detection of first generation migrants only. Assumes sexual reproduction	Paetkau et al. (2004), Piry et al. (2004)	–	–
Population size				
BOTTLENECK	Infinite allele model, stepwise mutation model, or two-phase mutation model	Piry et al. (1999)	Reports on sensitivity to mutation model conflict	Cornuet and Luikart (1996), Leblois et al. (2006), Peery et al. (2012)
BOTTLENECK	Infinite allele model, stepwise mutation model, or two-phase mutation model	Piry et al. (1999)	Low power of test may limit their utility	Peery et al. (2012)
*M*-ratio	Generalized stepwise mutation model	Garza and Williamson (2001)	Can significantly overestimate bottlenecks when mutation parameters are improperly specified	Peery et al. (2012)
*M*-ratio	Generalized stepwise mutation model	Garza and Williamson (2001)	Low power of test may limit their utility	Peery et al. (2012)
MSVAR	Stepwise mutation model	Beaumont (1999)	Reports on sensitivity to mutation model conflict	Girod et al. (2011), Faurby and Pertoldi (2012)

Piry et al. (2004) developed GENECLASS2, which uses a Monte Carlo resampling algorithm to identify migrants using genetic distance, allele frequencies, and Bayesian criteria. In contrast to BayesAss, methods in GENECLASS are derived for detection of migration rate and migrant individuals in the first generation only (Paetkau et al. 2004). GENECLASS2 utilizes reference subpopulations to assign unknowns to reduce identification of false migrants and is designed to reduce bias caused by unequal sample sizes (Piry et al. 2004). However, GENECLASS2 assumes that the species under study is undergoing sexual reproduction (Piry et al. 2004). BIMr is a program recently developed by Faubet and Gaggiotti (2008) that implements the *F*-model and therefore can estimate migration rate and detect migrants at a lower level of population differentiation compared to BayesAss or GENECLASS2. In addition, BIMr can analyze the influence of environmental variables on migration rate (Faubet and Gaggiotti 2008). In summary, while assignment-based methods that estimate migration rate such as BayesAss, GENECLASS2, and BIMr are effective, their application is specialized based on sampling scheme requirements and narrow assumptions of migration and differentiation (Piry et al. 2004; Faubet and Gaggiotti 2008; Broquet et al. 2009; Meirmans 2014).

## Population Size

The size of a subpopulation is a parameter central to population genetics because trends of expansion or declines in population size can be informative about an organism's demographic history and future trajectories. Due to various factors that limit the reproductive contribution of a given individual to the next generation (Leberg 2005; Charlesworth 2009), the actual parameter of interest commonly is effective population size (*N*_e_), or the “number of individuals in an ideal population that would lose genetic variation at the same rate as the actual population” (Crow and Kimura 1970; Leberg 2005). *N*_e_ governs the rate that genetic drift acts on a subpopulation, which is described in its relationship to the scaled rate parameter Θ = *xN*_e_*μ*, where *x* is the inheritance scalar and *μ* is the mutation rate. In practice, therefore, *N*_e_ is crucial in conservation and ecology because it provides measurement and warning of the conservation status of a given organism. It is not feasible to determine *N*_e_ by observation (Vucetich and Waite 1998), but genetic data can be used to estimate *N*_e_. Methods for estimating *N*_e_ vary depending on the timescale of interest, ranging from current to ancient *N*_e_, and use of parameters ranging from heterozygosity to Θ, respectively. Estimators for recent timescales are classified into inbreeding or variance effective population sizes, which are based on a single sample or temporal samples (spaced over generations), respectively (Leberg 2005; Luikart et al. 2010). The background of *N*_e_ (Charlesworth 2009), the range of scales and appropriate *N*_e_ estimation methods for each (Wang 2005), practical considerations for estimating *N*_e_ (Leberg 2005; Palstra and Ruzzante 2008; Luikart et al. 2010; Tallmon et al. 2010; Waples and Do 2010; Barker 2011; Peel et al. 2013), and the biases of temporal estimation (Hoehn et al. 2012; Ryman et al. 2014) have been thoroughly reviewed.

A BOTTLENECK, or a reduction in *N*_e_, leads to an excess of common microsatellite alleles compared to rare alleles than would be expected under equilibrium (Cornuet and Luikart 1996). Instead of making point estimates of *N*_e_, several methods are available that infer past changes in *N*_e_. Cornuet and Luikart (1996) exploit the differential influence of rare alleles on two estimates of expected heterozygosity, based either on Hardy–Weinberg or mutation–drift equilibrium, in the heterozygosity-excess test for population bottlenecks. BOTTLENECK (Piry et al. 1999) is the most widely used program for performing heterozygosity-excess tests (Peery et al. 2012). Garza and Williamson (2001) also utilize rare alleles as a means to identify bottlenecks in their statistical test based on *M*, the mean ratio of number of alleles to allele size range, and the GSMM. MSVAR, developed by Beaumont (1999) and Storz and Beaumont (2002), is a likelihood-based method that infers population size changes based on several model parameters using microsatellites following the SMM. Major assumptions and problems of these methods are summarized in Table 6.

The performance of the heterozygosity-excess/BOTTLENECK, *M*-ratio, and MSVAR methods has been compared in extensive evaluations (Williamson-Natesan 2005; Girod et al. 2011; Peery et al. 2012). Among their simulations, BOTTLENECK detected nearly 60% of simulated population expansions but only less than 10% of simulated declines (Girod et al. 2011). However, the heterozygosity-excess method is more accurate for recent and mild bottlenecks than the *M*-ratio test (Williamson-Natesan 2005). Although the *M*-ratio test identified over half of the population contractions simulated by Girod et al. (2011), the *M*-ratio test is generally less accurate when contractions were recent or not severe (Williamson-Natesan 2005; Girod et al. 2011; Peery et al. 2012). MSVAR outperformed BOTTLENECK and *M*-ratio by detecting approximately 70% of simulated expansions and contractions, even under modest departures from the SMM (Girod et al. 2011). Regarding MSVAR, Girod et al. (2011) also note that parameter estimation was more accurate under contractions compared to expansions and discuss significant differences in methods between different versions of MSVAR. The *M*-ratio test can significantly overestimate bottlenecks when the multistep mutation parameter is not properly specified (Peery et al. 2012). While the heterozygosity-excess test was reported to be relatively insensitive to violations of the mutation model (Peery et al. 2012), others have shown that tests for declines with BOTTLENECK are confounded, as observed by Girod et al. (2011), when microsatellite loci closely follow the SMM versus the IAM or GSMM (Cornuet and Luikart 1996; Leblois et al. 2006). MSVAR has been reported to be confounded by deviations from the SMM (Faurby and Pertoldi 2012). Although the *M*-ratio outperformed the heterozygote-excess test, Peery et al. (2012) conclude that the low power of both tests to detect population declines limits their utility and that when declines are detected, inferring the timing of these events is problematic. The practice of sampling more loci or individuals to increase the power of these two tests is under debate (Hoban et al. 2013b; Peery et al. 2013).

In addition to the program NeEstimator that allows *N*_e_ inference by multiple approaches in an easily accessible interface (Do et al. 2014), several new methods for estimating *N*_e_ have recently become available. Due to their analyses of family relationships, kinship-based analyses contain information about *N*_e_ (Waples and Waples 2011). Wang (2009) developed the sibship assignment (SA) method to estimate *N*_e_. Implemented in COLONY2, it is robust to violations of some common assumptions, such as random mating, and outperformed methods based on heterozygosity-excess or temporal sampling (Wang 2009). Most methods of *N*_e_ estimation assume discrete generations, and acknowledging and accounting for overlapping generations is a significant issue for inference of *N*_e_ (Wang 2009; Luikart et al. 2010). The estimator by parentage assignment (EPA) method, implemented in AgeStructure, enables proper estimation of *N*_e_ for species with overlapping generations if the age and sex of individuals are known (Wang et al. 2010). Other methods for estimating *N*_e_ in datasets with overlapping generations are available (Coombs et al. 2012; Jorde 2012). Tallmon et al. (2008) used an approximate Bayesian computation (discussed below) approach in ONeSAMP that uses eight summary statistics to infer *N*_e_, but this method is restrictive with respect to missing data (Peel et al. 2013). Some of these methods have not yet been formally evaluated, but they have been compared in empirical studies (Barker 2011; Skrbinšek et al. 2012). For historical or longer timescales, several estimators of Θ are available (Xu and Fu 2004; RoyChoudhury and Stephens 2007; Haasl and Payseur 2010). Using a novel theoretical understanding of expected allele frequencies for microsatellites, Haasl and Payseur (2010) developed three new estimators of Θ specifically for microsatellites. While these new estimators have lower error than previous Θ estimators, Haasl and Payseur (2010) note that, like for *N*_e_, no estimator performed the best in all conditions. Moreover, when comparing estimators, Haasl and Payseur (2010) found that the outlier value was generally the most representative of the true parameter.

Investigating recent population size using microsatellites is challenging. Results from different methods can vary widely (Barker 2011), partially because they can be focused on estimating *N*_e_ or changes in *N*_e_ during different periods of time in the past (Leberg 2005; Wang 2005; Charlesworth 2009; Luikart et al. 2010). For example, the number of loci and samples typical of most studies is not sufficient to distinguish moderate and large population sizes (500 < *N*_e_ < 5000) (Luikart et al. 2010; Antao et al. 2011). In addition, most methods of *N*_e_ inference assume simple population models, but factors such as migration, asymmetrical migration, spatial structuring, and reproductive variance can confound inference of *N*_e_ (Broquet et al. 2010; Chikhi et al. 2010; Waples 2010; Waples and England 2011; Hoban et al. 2013c; Paz-Vinas et al. 2013). Another confounding factor is the proposal that microsatellite mutation rate is proportional to the distance between heterozygous allele pairs (Amos et al. 1996, 2008; Amos 2011; Masters et al. 2011). Because it suggests a link between *N*_e_ and mutation rate, this observation has potentially significant implications for the inference of demographic history using microsatellite loci (Amos et al. 2008; Amos 2010) and also nucleotide sequences (Amos 2013). Therefore, research methods should be carefully crafted to address questions of interest and results interpreted with extreme caution (Barker 2011). In particular, sampling size and scheme, and the number and information content of microsatellites should be designed to address the timescale and objectives (Luikart et al. 2010; Tallmon et al. 2010; Waples and Do 2010; Antao et al. 2011; Peery et al. 2012; Hoban et al. 2013a).

## Evolutionary History

In population genetics, investigation of the evolutionary history of an organism can include inference on timescales all the way back to the speciation event for that organism. Compared with simpler methods for which multiple scenarios can give rise to similar patterns, historical inference methods have the advantage of jointly estimating multiple parameters to disentangle the competing influences of different evolutionary processes (Marko and Hart 2011). A brief overview of some of these relevant methods is given in Appendix S7. Microsatellites are often rejected for the purposes of historical inference, but they can be used (Sun et al. 2009; Bird 2012) if loci are well behaved or understood. The mutation rate and model are often critical considerations due to the depth of inference possible with these methods. Therefore, like other methods above, here we discuss methods for ancestral inference mostly in the context of the microsatellite mutation model.

### Coalescent estimation

Historical inference using the coalescent is performed by sampling genealogies back in time to the common ancestor of the sample under study (Appendix S7). The program MIGRATE (Beerli and Felsenstein 1999) contains support for a “ladder” model of microsatellite mutation, which appears similar to the GSMM, and a Brownian motion model, which is a simplified version of the ladder model that facilitates faster computation (Kuhner 2006). In addition to the SMM and the Brownian models, the program LAMARC includes support for the K-allele model and a combined K-allele/SMM model (Kuhner 2006). The isolation with migration (IM) programs contain support for the SMM only (Hey et al. 2004), whereas Bayesian Evolutionary Analysis by Sampling Trees (BEAST) relaxes some of the common constraints with microsatellites by integrating over a variety of microsatellite mutation models (Wu and Drummond 2011). All four of these methods allow mutation rate to vary among loci or to be specified on a per-locus basis.

Difficulties related to long run times and achieving convergence (Appendix S7) are amplified when analyzing microsatellite data because computation is considerably slower for the SMM compared to the Brownian motion model (Kuhner 2006) or the IAM (Hey 2011). Indeed, the SMM was added to the IM line of programs only for the purpose of analyzing microsatellites in conjunction with closely linked SNPs in their flanking regions (Hey et al. 2004). The current implementation of analyzing microsatellites with BEAST is likely to be slow for large datasets (Wu and Drummond 2011). BEAST allows missing data, whereas IM does not.

Many studies employing IM programs use microsatellites in conjunction with organelle or nuclear DNA sequences. Even when these different markers are combined, the lack of sufficient information can prevent convergence (Limborg et al. 2012) or force parameters to be removed from the model (Kyrkjeeide et al. 2012). Several studies have analyzed datasets consisting of solely microsatellites using the IM line of programs (e.g., Buonaccorsi et al. 2011; Charpentier et al. 2012; Kondo et al. 2012; Portnoy and Gold 2012; Roy et al. 2012), but these data were generally highly informative. Conversely, too many loci can drastically slow down the algorithm (Hey and Nielsen 2004) and require only subsets of loci to be used (Buonaccorsi et al. 2011). To our knowledge, the use of IM, LAMARC, or MIGRATE with solely microsatellite data has not been formally investigated. However, the sensitivity of IM programs to violations of various assumptions for other mutation models has been studied in detail (Strasburg and Rieseberg 2010, 2011; Sousa et al. 2011). The behavior of MIGRATE under select circumstances has also been discussed (Beerli 2004, 2006, 2007; RoyChoudhury and Stephens 2007). Performance of these methods with microsatellite data is likely to vary on a case-by-case basis, and users are recommended to thoroughly ensure convergence of analyses and/or consult with a colleague that has experience with these programs.

### Approximate bayesian computation

As mentioned at several points in this review, performing simulations is highly recommended to ensure that inferences being drawn from the observed data are reflective of the true population demography. Programs and methods for generating simulated data have been reviewed (Epperson et al. 2010; Hoban et al. 2012). Simulations and analysis of simple summary statistics have been formalized into the approximate Bayesian computation (ABC) statistical framework (Appendix S7) (Beaumont et al. 2002; Beaumont 2010). An infrequently addressed topic with respect to microsatellites is mutation model. The programs popABC (Lopes et al. 2009), abc (Csilléry et al. 2012), and ABCtoolbox (Wegmann et al. 2010) lack flexibility to define microsatellite mutation models, whereas DIYABC includes support for the GSMM to allow for mutations of more than one repeat unit and for defining size constraints (Cornuet et al. 2010). EggLib, a comprehensive data handling and population genetic analysis Python package that includes ABC capability, contains options for the IAM, SMM, and a two-phase model, and to weight loci for probability of mutation (De Mita and Siol 2012). Support for additional models may allow more closely fitting approximations, but may be an unnecessary overparameterization at the expense of other parameters of interest (Cornuet et al. 2006).

## Conclusions

Microsatellites can be powerful tools for inferring population patterns and processes in both human biology and non-human systems and continue to be widely used in the literature. However, the very properties that confer advantages to microsatellites in this role also can confound inference using methods common to population genetics. Due to the perceived convenience and power of microsatellite markers, inferences may be gleaned that exceed the capabilities of the methods or sample at hand. We find that, in general, methods used to analyze microsatellite data have not been thoroughly evaluated under conditions near the edge of the theoretical envelope. Moreover, several methods lack formal validation with microsatellites and should be used with extreme caution. This review lacks a comprehensive list of methods or programs and omits discussions on other difficult problems with respect to microsatellites that include gametic linkage disequilibrium and recombination (see Gompert and Buerkle 2013 for a detailed review), network inference, distance calculations, and selection (Haasl and Payseur 2013). However, here we have provided a synthesis of the weaknesses of microsatellites that we hope researchers will use to guard against exceeding the limitations of these markers in population genetics. Researchers analyzing microsatellite datasets are encouraged to perform simulations to assist study design and marker development (De Mita and Siol 2012; Karl et al. 2012; Hoban et al. 2013a), and as confirmation, that methods are performing as expected (Pearse and Crandall 2004; Dufresne et al. 2014). For the first time users, simulations provide experience with computational and programming techniques prior to analyzing the actual dataset. Integrating as many methods as possible to answer relevant questions is a powerful approach due to complementary or differential interactions (Garrick et al. 2010).

## Data Availability

Search strings used to download records from Web of Science and scripts used to analyze trends in the literature are available at the Dryad Digital Repository (http://dx.doi.org/10.5061/dryad.j6567).

## Supporting Information

**Appendix S1.** Spatial considerations. **Appendix S2.** Exploratory methods. **Appendix S3.** Descriptive statistics. **Appendix S4.** Overview of model-based clustering methods. **Appendix S5.** Model-based *K* inference. **Appendix S6.** Summary of use of descriptive statistics for inferring migration. **Appendix S7.** Overview of methods for ancestral inference.
