# Supplementary material for: Challenges in analysis and interpretation of microsatellite data for population genetic studies
Source: Ecol Evol. 2014 Oct 30;4(22):4399–428. doi: 10.1002/ece3.1305 (PMC4267876; doi:10.1002/ece3.1305)
Supplement: Supplementary file 1 [file ece30004-4399-SD1.docx]

SUPPLEMENTARY MATERIAL

Challenges in Analysis and Interpretation of Microsatellite Data for Population Genetic Studies

Alexander I. Putman* and Ignazio Carbone

Department of Plant Pathology, North Carolina State University, Raleigh, NC 27695-7616

*Corresponding author (aiputman@ncsu.edu; +1 919 438 3810)

Table of Contents

Appendix S1: Spatial Considerations 2

Appendix S2: Exploratory methods 4

Clustering 4

Ordination 6

Admixture Inference 9

Use of Clustering and Ordination 10

Appendix S3: Descriptive Statistics 10

Fixation Statistics 10

Diversity-Based Statistics 13

Debate on Descriptive Statistics 15

Appendix S4: Overview of model-based clustering methods 17

Admixture 19

Gametic Linkage 20

Detecting Weak Structure 21

Relatedness 23

Null Alleles 24

Appendix S5: Model-based K inference 25

Ad-Hoc Methods 25

Formal Inference 27

Appendix S6: Summary of use of descriptive statistics for inferring migration 28

Appendix S7: Overview of methods for ancestral inference 31

Coalescent Estimation 31

Approximate Bayesian Computation 33

Appendix S1: Spatial Considerations

When delimiting subpopulations that are weakly differentiated is a study objective, the subpopulations of interest may exist in some degree of contact or in a cline. Therefore, the inclusion of spatial information into parametric inference may improve detection of subpopulations. Programs that can perform spatially-explicit (i.e., include spatial information for each individual) inference of population structure include BAPS (Corander *et al.* 2008b), GENELAND (Guillot *et al.* 2005; Guillot *et al.* 2012), and TESS (Chen *et al.* 2007; Durand *et al.* 2009b). These programs, their implemented models, and their performance have been previously reviewed (Chen *et al.* 2007; François & Durand 2010; Guillot *et al.* 2009) and debated (Durand *et al.* 2009a; Guillot 2009a, b). In general, models without admixture have constraints that are too strict to allow investigation of populations in contact or in clines (François & Durand 2010). While admixture models in these programs are more useful at delimiting subpopulations in close proximity and accurately inferring the number of clusters, some admixed individuals may be assigned to incorrect cluster(s) (François & Durand 2010). Even though STRUCTURE does not include a spatially explicit model, STRUCTURE and the spatial methods GENECLUST, GENELAND, and TESS performed well at detecting a cline of allele frequencies (Chen *et al.* 2007; François & Durand 2010). In contrast, Schwartz and McKelvey (2008) found that STRUCTURE was confounded by diversity gradients. Chen et al. (2007) reported that TESS is most efficient at identifying contact between subpopulations having a low level of differentiation. In addition to model-based methods, spatial principal component analysis (sPCA) is a spatially explicit ordination method (Jombart *et al.* 2008). In its development, sPCA was evaluated on simulated microsatellite data under a variety of demographic scenarios (Jombart *et al.* 2008), but under singular migration and mutation rates. sPCA has been employed on numerous microsatellite datasets since its release, but interpretation of spatial analyses of PCA should be performed with caution (DeGiorgio & Rosenberg 2013; François *et al.* 2010; Novembre & Stephens 2008). Other methods such as geographically weighted PCA (reviewed by Harris *et al.* 2011) are available, but have not been applied to microsatellites to our knowledge. The Population Graph method, which uses graph theory and is model-free (Dyer & Nason 2004), is available in the software suite GeneticStudio (Dyer 2009) to perform spatially explicit genetic analyses, but has not been widely used or studied.

Inference of population structure and migration is also of central interest to landscape genetics, a rapidly expanding area of research that studies population genetics in a spatial context to identify the landscape features potentially responsible for the observed genetic patterns (Holderegger & Wagner 2008; Manel & Holderegger 2013). While developed as a program for landscape genetics, GENELAND includes a non-spatial model, and like STRUCTURE, models for admixture, correlated allele frequencies, null alleles, and phenotype information (Guillot 2008; Guillot *et al.* 2005; Guillot *et al.* 2012; Guillot & Santos 2009; Guillot *et al.* 2008). However, the phenotype model in STRUCTURE is not analogous to that for GENELAND (Guillot *et al.* 2012), and evaluations of this program have focused on the spatial models. These spatial methods in landscape genetics for estimating migration have been reviewed (Anderson *et al.* 2010; Manel & Holderegger 2013; Segelbacher *et al.* 2010; Storfer *et al.* 2007) and evaluated (Blair *et al.* 2012; Dyer *et al.* 2010; Jaquiéry *et al.* 2011; Landguth *et al.* 2010a; Landguth *et al.* 2010b; Safner *et al.* 2011).

Appendix S2: Exploratory methods

Clustering

Determination of the optimal or statistically significant number of clusters and assignment of data points to clusters are broad problems that have received considerable attention from diverse disciplines (Filippone *et al.* 2008; Jain *et al.* 1999; Xu & Wunsch 2005). In genetics, cluster analysis has been extensively applied to microarray data, and more recently genome analyses (reviewed by Jay *et al.* 2012; Thalamuthu *et al.* 2006; Xu & Wunsch 2005). Cluster analysis has been traditionally applied to population genetics for exploring multilocus data, but has recently experienced a broadening of interest.

As in other disciplines, a robust determination of the number of clusters (*K*) given the observed data is central to inference of population structure. In the extensive field of cluster analysis, there are many methods to accomplish this task, broadly categorized as indices called stopping rules or as distribution-fitting techniques (Milligan & Cooper 1985; Xu & Wunsch 2005). For example, Milligan and Cooper (1985) performed a general evaluation of 30 indices and found that the Calinski-Harabasz index (Calinski & Harabasz 1974) generally performed the best. Indeed, the Calinski-Harabasz index performs well in population genetic analyses according to anecdotal reports (Meirmans 2011a), is implemented in several programs, and has been utilized in numerous studies (Atallah *et al.* 2010; Dufresne *et al.* 2011; Goss *et al.* 2009). The gap statistic appears to be less frequently used for microsatellite data and has been reported to underperform relative to other cluster determination methods (Lee *et al.* 2009; Meirmans). The gap statistic is characterized as performing best when clusters are well separated and the number of clusters is small (Galluccio *et al.* 2012). Methods that evaluate the fit of a distribution to the data, such as Akaike’s information criterion (AIC) and Bayesian information criterion (BIC), can also be used to infer *K* (Fraley & Raftery 1998). BIC in particular has gained recent favor, is available in R packages such as *bayesclust* (Gopal *et al.* 2012) and MCLUST (Fraley & Raftery 2003), and has been reported to perform best on population genetic datasets using SNPs (Jombart *et al.* 2010; Lee *et al.* 2009).

Like *K* optimization, there are numerous algorithms for assigning individuals to clusters (reviewed by Xu & Wunsch 2005) that include methods such as distribution- and density-based clustering. However, two types are commonly employed in population genetic studies. The first is hierarchical, in which data points are connected at various levels according to their distance (Xu & Wunsch 2005). One of these distance linkage methods is unweighted pair group method with arithmetic mean (UPGMA) (Sokal & Michener 1958), which is popular with microsatellite datasets. Ward (1963) is another clustering method that has been shown to be effective in detecting structure in germplasm collections (Odong *et al.* 2011). Hierarchical methods are useful because by definition they account for and allow visualization of multiple levels of structure in the data. However, plots of hierarchical analysis results can become confusing as dataset complexity increases, thereby hampering interpretation. Another limitation of UPGMA is that it cannot depict non-hierarchical structure (Kalinowski 2009, 2011). Neighbor joining (NJ) is a clustering method that was developed for inferring phylogenetic trees using a method similar to minimum evolution (Felsenstein 2004). A major difference between UPGMA and NJ is that UPGMA outputs rooted dendograms because the rate of evolution is constant across all branches of the tree, whereas NJ allows for the molecular clock to vary among branches and therefore produces phylograms (Felsenstein 2004).

Centroid clustering assigns data points to an assumed number of *k* clusters based on their distance from the center, and in contrast to hierarchical clustering, produces only a single level of classification. The most popular approximation method is *k*-means. Drawbacks to *k*-means include need for an assumed *k* value and the possibility of settling on local optima (Galluccio *et al.* 2012; Xu & Wunsch 2005). For each algorithm developed for centroid clustering, a large number of algorithmic variants and derivatives exist that attempt to address the respective shortcomings of each type, such as for *k*-means (Xu & Wunsch 2005).

No clustering algorithm or method for determining *K* is optimal for every data set (Jain *et al.* 1999; Xu & Wunsch 2005). For datasets with noise, outliers, or complicated structure, attempting analysis with multiple algorithms is recommended. Mainstream programs commonly incorporate multiple algorithms (Morris *et al.* 2011), and others develop formal procedures for combining results from multiple algorithms (Albatineh & Niewiadomska-Bugaj 2011; Fraley & Raftery 2003; Mimaroglu & Aksehirli 2011). Following cluster analysis, cluster validation is an important step because many methods have not been tested extensively enough and do not provide a means to evaluate the significance of their results (Handl *et al.* 2005). Cluster validation has been discussed elsewhere (Handl *et al.* 2005; Xu & Wunsch 2005), and is available in numerous packages (e.g., Brock *et al.* 2008).

Ordination

A population genetic dataset consisting of many loci and individuals may be reduced into a few uncorrelated variables by methods called ordination in reduced space, or simply ordination, which are a subset of multivariate analysis (Jombart *et al.* 2009). Ordination has broad applicability across disciplines, and has a long history of use in genetics (Cavalli-Sforza 1966; Menozzi *et al.* 1978). In contrast to some statistics discussed below, such as fixation statistics, ordination methods are exploratory because they summarize the data while not depending on assumptions such as Hardy-Weinberg or gametic linkage equilibrium (Jombart *et al.* 2009). These methods are also computationally fast, making them ideal for analyzing extremely large and complex datasets. Briefly, these methods construct principal axes in the data, about which dispersion, or inertia, is maximized. Eigenvalues represent the variance about each principal axis (Lee *et al.* 2009). The relationship of data points to these principal axes is defined by its principal components. Jombart et al. (2009) provided an extensive review on ordination and included common mistakes and basic recommendations for population genetic analysis.

Principal component analysis (PCA) (Cavalli-Sforza 1966; Pearson 1901) summarizes variance in the data while retaining distance information between alleles and is the simplest ordination method applied to population genetics. For instance, because the frequency of a given allele is binomial, variance is generally highest for frequencies close to 0.5 and generally lowest for frequencies close to 0 or 1. Therefore, PCA can be biased toward alleles with frequencies near 0.5 and confound inferences of population structure (Jombart *et al.* 2009).

In contrast, principal coordinate analysis (PCoA; sometimes referred to as PCO) does not depict alleles but instead decomposes a previously-calculated measure of distance or differentiation (Jombart *et al.* 2009). In addition to average square distance (Bird 2012; Sun *et al.* 2009), allele sharing distance is a common measure used in population genetics that is believed to perform well for microsatellites (Gao & Martin 2009) and has been mostly used to create NJ trees (Bowcock *et al.* 1994; Koskinen 2003; Sodhi *et al.* 2008). However, allele sharing distance of microsatellites has also been used to initiate PCoA (Meece *et al.* 2011; Wadl *et al.* 2008). In addition, tables of pair-wise measurements of differentiation such as *F_ST_* have been analyzed by PCoA (Zhivotovsky *et al.* 2003). Thus, PCoA depends on the assumptions of the model employed to calculate distance or differentiation, and is subject to the nuances of the chosen statistic and estimator.

Different ordination methods may be performed in successive steps to overcome the limitations of each. In the first step of discriminant analysis of principal components (DAPC) (Jombart *et al.* 2010), PCA is performed to summarize diversity among individuals. After individuals are assigned to groups using *k*-means clustering and the number of groups is determined using BIC, discriminant analysis (DA) is performed on the decomposed data to assess differentiation among groups by partitioning diversity into within- and between-group components. Group assignment is an independent step in the analysis, therefore any desired clustering method may be used (Jombart *et al.* 2010). DAPC, available in the R package *adegenet* (Jombart 2008), performs well under various population genetic models (Jombart *et al.* 2010), but it can be confounded by isolation by distance (Blair *et al.* 2012).

Ordination methods and advanced cluster analyses are particularly advantageous for extremely large and high-dimensional datasets, such as microarray studies on thousands of genes, or genomic or population genetic studies with tens of thousands of SNP loci. SNPs have only recently been incorporated into an ordination framework using appropriate genetic distances. Patterson et al. (2006) developed an algorithm to determine the statistical significance of eigenvectors obtained from analysis of SNP data using a modified form of PCA. While Patterson et al. (2006) note that it may be used with microsatellite data, the robustness of their algorithm on microsatellite data is unclear. There are several other methods that formally incorporate genetic data in ordination analysis of dominant markers (Reeves & Richards 2009) and SNPs (Gao & Starmer 2008; Intarapanich *et al.* 2009; Lee *et al.* 2009; Limpiti *et al.* 2011; Ma & Amos 2012). A method using spectral hierarchical clustering with iterative pruning has been proposed, and although it was developed for SNPs, the software package can accept any pairwise similarity matrix as input (Bouaziz *et al.* 2012).

Admixture Inference

Admixture, in which portions of the genome are derived from different populations, can be visually detected in PCA graphs because it is one possible cause of the appearance of individuals along a line between two parent subpopulations (McVean 2009; Patterson *et al.* 2006). Diversity that is continuous along time or space can also cause clines to appear in PCA results (Jombart *et al.* 2010). Under these conditions, however, assigning an individual to only a single genetic cluster is an inaccurate representation and can confound inference of population structure unless explicitly accounted for in the model. In situations of admixture or diversity gradients, objectively assigning individuals to clusters is less straightforward because all of the clustering methods discussed above use hard clustering algorithms that assign each point to a single cluster (Xu & Wunsch 2005). Fuzzy or soft clustering allows for partial cluster membership and may facilitate accurate population genetic inference in the presence of admixture. Lee et al. (2009) evaluated the fuzzy method soft *K*-means and found it useful in comparison to hard clustering methods and model-based structure inference. Fuzzy methods are intensively studied in other disciplines, but despite their potential, their application to population genetic studies has been limited. However, Ma and Amos (2012) recently developed a modified PCA for SNPs that incorporates mixed ancestry, allowing formalized inference of admixture. Analysis and interpretation of admixture has also been described in a genealogical framework (McVean 2009).

Use of Clustering and Ordination

Commonly, ordination results are used to confirm the results of model-based analyses by visual comparison (Reeves & Richards 2009). Ordination results may also be used to visually estimate the number of *K* subpopulations, which in turn is used as input for population-assignment methods that require an assumed *K* value (Intarapanich *et al.* 2009). Visual interpretation of PCA results may be confounded by unequal sample sizes (Ma & Amos 2012), but software packages to enhance visualization of complex results are available (Rajaram & Oono 2010). In other disciplines, cluster analysis is generally performed on raw data, and ordination can be used prior to clustering to reduce the complexity of an intractably large dataset. However, with the exception of UPGMA and neighbor joining, application of clustering techniques to population genetic datasets has been limited. To increase objectivity of inferring *K*, clustering analysis may be performed on PCA output (e.g., Gao & Starmer 2008; Hausdorf & Hennig 2010; Jombart *et al.* 2010; Liu & Zhao 2006; Reeves & Richards 2009).

Appendix S3: Descriptive Statistics

Fixation Statistics

Wright’s *F_ST_* is a parameter that measures “the extent to which the process of fixation has gone toward completion” in a subpopulation relative to the entire population (Wright 1978), and is among a group of *F*-statistics based on identity by descent that were derived to detect inbreeding (Wang 2012b; Wright 1943). Because it partitions variation among defined groups, use of *F_ST_* has been co-opted into an identity by state measure to quantify the level of differentiation among subpopulations and has become one of the most widely used statistics in genetics (Holsinger & Weir 2009; Leng & Zhang 2011; Wang 2012b). The use of these statistics for describing population structure has been thoroughly reviewed (Holsinger & Weir 2009; Meirmans & Hedrick 2011; Wang 2012b), but here we provide a brief synopsis of this topic with a focus on microsatellites.

Because it was originally derived for biallelic data and suffers from sampling issues (Holsinger & Weir 2009; Meirmans & Hedrick 2011; Whitlock 2011), numerous estimators of *F_ST_* (reviewed by Balding 2003) and *F_ST_*-like indices (collectively, *F*-statistics) have been developed to address some of these limitations. *θ* (Weir & Cockerham 1984; Weir & Hill 2002) is a widely used method of moments estimator of *F_ST_* calculated using an analysis of variance (ANOVA) of allele frequencies to account for sampling (Holsinger & Weir 2009). To utilize the *F_ST_* framework for multiallelic markers like microsatellites, Nei (1973) proposed the statistic *G_ST_* to quantify genetic differentiation among subpopulations. High levels of diversity artificially depress the maximum possible *F_ST_* (Jakobsson *et al.* 2013) and *G_ST_* (Hedrick 2005) value.

The above parameters are derived assuming the IAM. To help ameliorate problems due to uncertainty in mutation model and rates, two parameters were developed to account for the SMM. These two parameters are relatives of *F*-statistics in that they represent allelic differentiation to some degree, but because they account for a particular mutation model they in addition represent the evolutionary distance among alleles (Holsinger & Weir 2009). Because microsatellite alleles are known to occur in a finite range, however, this distance interpretation should be made with caution (Balloux & Lugon-Moulin 2002). The first and most widely-cited parameter is *R_ST_*, which was first reported by Chakraborty and Nei (1982) and later formalized by Slatkin (1995). Rousset (1996) derived the second parameter, ρ*_ST_*. Based on the parameters’ comparison of alleles drawn from either the entire population or different subpopulations, *R_ST_* is considered an analogue to *G_ST_* for microsatellites, whereas ρ*_ST_* is considered a microsatellite analogue for *F_ST_* (Estoup & Angers 1998; Michalakis & Excoffier 1996; Rousset 1996).

Parameters that include allele size are typically associated with high variance (Balloux & Goudet 2002; Gaggiotti *et al.* 1999; Slatkin 1995), which may lead to biased estimates of population differentiation because loci with extreme levels of variance will make a disproportionate contribution to the overall population differentiation (Goodman 1997). Therefore, like Weir and Cockerham’s *θ*, microsatellite parameters are most often estimated in an analysis of molecular variance (AMOVA) framework (Excoffier *et al.* 1992), which has been extended to microsatellites using a generalized weighting scheme to account for differences and interactions among loci (Michalakis & Excoffier 1996). Meirmans (2012) investigated the AMOVA framework further to show that it is related to *k*-means clustering, and developed methods to perform both simultaneously. As a special case of Michalakis and Excoffier’s (1996) estimator, Goodman (1997) proposed that data be standardized to the sample mean of each locus. This standardized data reduces the variance in estimating ρ*_ST_* and also allows comparisons among different loci (Goodman 1997, see also Balloux & Goudet 2002; Meirmans & Hedrick 2011) for discussions).

*F*-statistics were derived from the infinite island model of population structure, in which an infinite number of subpopulations of identical size and having independent allele frequencies are exchanging migrants at equal rates (Meirmans & Hedrick 2011; Song *et al.* 2006). A single value of *F_ST_* adequately describes total population structure under these conditions (Gaggiotti & Foll 2010). Empirical studies often estimate pair-wise values of *F_ST_* among sampling populations because these migration assumptions are rarely met in practice (Gaggiotti & Foll 2010; Slatkin 1993). Alternatively, *F_ST_* values specific to each subpopulation may be estimated using methods such as an extension of the *θ* estimator (Weir & Hill 2002) or the *F*-model, which essentially is a relaxed island model allowing for unequal subpopulation sizes and migration rates (Gaggiotti & Foll 2010). Currently, however, the *F*-model does not account for hierarchical population structure and assumes all migrants originate from a single pool (Gaggiotti & Foll 2010). For allele frequencies, correlation among populations due to shared ancestry (Balding 2003), migration (Fu *et al.* 2005), or because a finite number of subpopulations exchanging migrants undergo drift together (Song *et al.* 2006) can cause overestimation of *F*-statistics using previously applied methods (Fu *et al.* 2005; Fu *et al.* 2003). In contrast, exact moment calculations have been shown to accurately estimate *F*-statistics (Fu *et al.* 2003; Song *et al.* 2006), but this method has not yet been adapted for use in empirical studies (Fu *et al.* 2005; Song *et al.* 2011).

Diversity-Based Statistics

In light of the deficiencies of *F*-statistics such as dependence on mutation and gene diversity outlined above, Jost (2008) used an approach based only on allelic differentiation to develop *D*, an explicit differentiation measure. As described by Whitlock (2011): “*F_ST_* measures deviations from panmixia, while *D* measures deviations from total differentiation.” *D* provides more sensible results for differentiation (Meirmans & Hedrick 2011), and at all levels of genetic diversity. For example, in contrast to *F_ST_*, *D* accurately identifies differentiation when gene diversity is high or when subpopulations do not share any alleles (Jost 2008, but see Wang 2012b). *D* can be confounded by high mutation rates, but is much less sensitive to mutation rate when loci follow the SMM or when the mutation rate is much lower than the migration rate (Leng & Zhang 2011, 2013). *D* is therefore recommended for differentiation inference when mutation rates are unknown due to its distance-like properties (Leng & Zhang 2011). Additional applications of *D* include a measure of the relative influence of mutation versus migration (Whitlock 2011).

Entropy was originally derived for thermodynamics, but is a generally useful concept in complex systems that was applied by Claude E. Shannon (Shannon 1948a, b) to develop the broadly applicable field of information theory. Shannon’s entropy, *^S^H*, is a diversity index that is the most widely used measurement of diversity in ecology and conservation, often for species surveys (Dewar *et al.* 2011; Sherwin 2010). Mutual information (MI) is used in information theory to quantify the interdependence of two variables (Dewar *et al.* 2011), and is a measure of differentiation in population genetics because it describes to what degree an individual’s genotype represents its subpopulation assignment (Sherwin 2010; Sherwin *et al.* 2006).

MI was found to have several advantages with respect to *F_ST_* when evaluating differentiation between two subpopulations under both the IAM and SMM. The index is unaffected by allelic richness, offers intuitive estimates of differentiation when subpopulations do not share alleles, accounts for unequal subpopulation sizes, allows for the possibility of more accurate estimates of migration and mutation model, and has increased sensitivity to rare alleles (Sherwin 2010; Sherwin *et al.* 2006). It should be noted that Sherwin et al. (2006) performed their simulations with a mutation rate of 10^-2^, which would likely strongly bias inference using fixation statistics. The use of *^S^H* in population genetic studies employing microsatellites is steadily increasing since becoming more accessible (e.g., Peakall & Smouse 2012) and entropy-based methods have also been cited as providing additional insight into population structure inference over other indices (Andrew *et al.* 2012; Blum *et al.* 2012). Despite its potential, the use of entropy in population genetics requires further investigation (Sherwin 2010; Sherwin *et al.* 2006).

Debate on Descriptive Statistics

Microsatellites are often employed to achieve high spatial or recent temporal resolution within populations that have not yet reached mutation-drift equilibrium (Anderson *et al.* 2011; Haasl & Payseur 2010; Lukoschek *et al.* 2008; Nauta & Weissing 1996; Takezaki & Nei 1996), but a poor understanding of the behavior of population parameters in these conditions could lead to erroneous interpretations of results (Leng & Zhang 2011, 2013). The following is a brief synopsis of debate regarding the relevance of *F*-statistics to theory and the application and interpretation of indices in population genetics.

*D* takes much longer to reach equilibrium under the stepwise mutation model compared to the infinite allele model (Leng & Zhang 2011). In addition, under non-equilibrium conditions, *D* and *G_ST_* are oppositely affected and to different degrees by the initial heterozygosity, but this effect depends on population size (Leng & Zhang 2011, 2013; Ryman & Leimar 2009). In practical applications such as for conservation, Lloyd et al. (2013) showed that *F_ST_*, *G’_ST_*, MI, and *D* were insufficient for detecting population structure between small and recently separated populations. Mutation rate and heterozygosity have a stronger influence in non-equilibrium conditions on *D* than *G_ST_* (Leng & Zhang 2011). In a one-dimensional stepping-stone model, similar values of *F_ST_* have different meanings across the range of geographic distance (Rousset 1996).

Estimators of *F_ST_* are excellent in describing structure and revealing demographic history, but only for markers having similar mutation rates or for subpopulations having similar levels of diversity or effective populations sizes (Holsinger & Weir 2009; Meirmans 2006; Meirmans & Hedrick 2011; Whitlock 2011). Using strict ranges of *F_ST_* values to interpret differentiation (e.g., value of 0-0.05 represents little differentiation) should be done carefully (Balloux & Lugon-Moulin 2002; Gregorius 2010; Jakobsson *et al.* 2013; Wright 1978). *θ* is a reliable estimator of *F_ST_* only when allele frequencies among subpopulations are not correlated (Song *et al.* 2006; Weir & Hill 2002).

*G_ST_* is an excellent measure of differentiation under certain conditions, but it can lead to underestimation (Heller & Siegismund 2009; Leng & Zhang 2011; Wang 2012b). Despite its derivation for mulitallelic data, *G_ST_* has been reported to represent differentiation only when two alleles are present (Gerlach *et al.* 2010). *G_ST_* has been suggested to be better for inference of migration than population structure (Jost 2009), but both *G_ST_* and *D* may still be appropriate differentiation measures in non-equilibrium conditions for modest mutation rates (Leng & Zhang 2013). The interpretability of *G_ST_* (Heller & Siegismund 2009; Whitlock 2011, but see Wang 2012b) or *G’_ST_* for demographic processes has been questioned (Leng & Zhang 2011; Ryman & Leimar 2009; Whitlock 2011). *D* is a superior measure of differentiation under some conditions, but it has no relevance to evolutionary theory because it is not a function of population size and therefore does not describe drift (Jost 2008, 2009; Leng & Zhang 2011; Meirmans & Hedrick 2011). *R_ST_* may be not be interpretable because of its sensitivity to deviations from the SMM and inferiority to *F_ST_* under some conditions (Balloux & Lugon-Moulin 2002; Gaggiotti *et al.* 1999), but not in others (Song *et al.* 2011). *θ* may better identify newly formed isolation following bottlenecks compared to *R_ST_* (Sefc *et al.* 2007).

Because *D* was only recently proposed and is still under development, its behavior and interpretability beyond answering questions of allelic differentiation is under active discussion (Heller & Siegismund 2009; Jost 2008, 2009; Leng & Zhang 2011, 2013; Ryman & Leimar 2008; Whitlock 2011). Additional discussions of fixation indices are available (Beaumont 2005; Edelaar & Björklund 2011; Edelaar *et al.* 2011; Gillet 2013; Gregorius 2010; Gregorius *et al.* 2007; Rousset 2013).

Appendix S4: Overview of model-based clustering methods

In model-based clustering, Bayesian methods are generally used to determine the probability of the data given the various parameters because the complexity of various models and the number of parameters employed precludes exact calculation. To do this, the first algorithm explores the parameter space (i.e., all permutations of all parameters) in discrete steps governed by the likelihood of the parameters (given the data) found at each step. A second algorithm samples from the steps of the first algorithm to construct a posterior probability distribution that serves as a representation of the true conditions. Model-based clustering methods for population genetics offer several genetic structure models for analysis, but also employ different searching algorithms that may have implications for interpreting results (Bohling *et al.* 2013).

STRUCTURE (Pritchard *et al.* 2000) is one of the most widely used programs in population genetic studies. When genetic population structure occurs, the total population has higher levels of gametic linkage disequilibrium than expected with random mating and higher levels of homozygosity (the Wahlund effect; Wahlund 1928) than expected under Hardy-Weinberg equilibrium (François & Durand 2010). STRUCTURE clusters individuals to maximize Hardy-Weinberg and gametic linkage equilibrium within subpopulations (Gao *et al.* 2007; Pritchard *et al.* 2000). STRUCTURE employs the Markov Chain Monte Carlo (MCMC) algorithm to explore the parameter space and Gibbs sampling to obtain the posterior probability distribution (Pritchard *et al.* 2000). While adhering to the same Hardy-Weinberg and gametic linkage equilibrium assumptions, the Bayesian Analysis of Population Structure (BAPS) software has employed several algorithms over its version history. Early versions used MCMC like STRUCTURE, but only when the dataset is too complex for direct enumeration (Corander *et al.* 2003), and later support for multiple simultaneous MCMC chains was added (Corander *et al.* 2004). Subsequent versions added features employing Bayesian predictive classification (Corander & Tang 2007; Corander *et al.* 2004) or greedy stochastic search algorithms (Corander & Marttinen 2006; Corander *et al.* 2006). Finally, further updates that improve computational efficiency and facilitate multithreaded applications have been added (Corander *et al.* 2008a).

In practice, the central difference among the algorithms found in these two programs is their convergence behavior. In general, MCMC algorithms have a tendency to converge on local maxima, whereas the algorithms implemented in newer versions of BAPS are designed to improve convergence on the best global solution (Corander *et al.* 2004). Additional practical implications that are reflective of difference among these algorithms are their speed (with BAPS being significantly faster), their ability to handle missing data (Corander & Tang 2007), and their applicability to estimating the number of *K* clusters in the data (Corander *et al.* 2006). Since its release (Pritchard *et al.* 2000), STRUCTURE has been appended and improved several times (Falush *et al.* 2003, 2007; Hubisz *et al.* 2009), and now includes at least 16 population structure models that can be selected based on options for admixture, linkage, inclusion of sampling information, or accounting for correlated allele frequencies. Some of these models have been reviewed by Gompert and Buerkle (2013), and Porras-Hurtado et al. (2013) provide a thorough treatment of the models and practical use of STRUCTURE. BAPS also includes several different population structure models, but whereas STRUCTURE uses the same computational algorithms for all options, BAPS employs different search strategies depending on model selection. BAPS and STRUCTURE employ similar types of ancestry models, the simplest of which clusters individuals into subpopulations. In the no-admixture model in STRUCTURE, all individuals are assumed to be members of one of *K* subpopulations (François & Durand 2010). In BAPS, this objective is achieved using the ‘clustering of individuals’ model (Corander *et al.* 2006).

Admixture

The second tier of models accounts for admixture. In the STRUCTURE admixture model (Pritchard *et al.* 2000), mixed ancestry from more than one of *K* ancestral, possibly unsampled, subpopulations leads to correlation among markers despite lacking physical association on the genome. For BAPS, however, admixture analysis is performed in a second step after clustering analysis due to the perceived complexity of jointly estimating admixture with the number of clusters and cluster assignment (Corander & Marttinen 2006). Thus, STRUCTURE is more prone to detect some low degree of admixture compared to BAPS (Bohling *et al.* 2013). The algorithm for admixture inference in BAPS is automatically selected based on the clustering method used. This is a conservative approach to avoid biased inferences for admixture when differentiation between subpopulations is low (Corander & Marttinen 2006). However, non-admixed individuals need to be included for BAPS to correctly infer admixture (François & Durand 2010). A non-model based method, FLOCK, was reported to be superior to STRUCTURE when the sample lacks non-admixed genotypes (Duchesne & Turgeon 2009).

Gametic Linkage

The admixture and no-admixture models in STRUCTURE and the ‘clustering of individuals’ model in BAPS assume that markers are physically unlinked (Pritchard *et al.* 2000). The assumption of linkage equilibrium is relaxed in STRUCTURE’s third ancestry model, the “admixture linkage disequilibrium” model, which accounts for additional correlation due to the loose physical linkage when large pieces of chromosomes are exchanged during admixture events. However, this model is not appropriate for markers that are tightly linked on relatively short distances from “background LD,” a third type of linkage disequilibrium that is caused by drift within subpopulations (Falush *et al.* 2003). Background LD may also be caused by the prevalence of the same allele combinations across more than one ancestral subpopulation (Falush *et al.* 2003). Despite the ability of this model to incorporate weak gametic linkage, Falush et al. (2003) recommend that an adequate portion of the data be derived from unlinked markers for accurate inference of population structure. In contrast, the ‘clustering with linked loci’ model in BAPS accounts for very tight linkage, like that, for example, which is found within loci from multi-locus sequence typing (Corander & Tang 2007).

The presence of ‘background’ gametic linkage disequilibrium can cause STRUCTURE to produce inflated estimates of *K* (Falush *et al.* 2003). Even if admixture is not suspected, other demographic events such as population bottlenecks can create a strong signature of linkage disequilibrium (Kaeuffer *et al.* 2007) and references therein). Sampling too few individuals per subpopulation when hierarchical structure is present may also cause a signal of linkage disequilibrium (Fogelqvist *et al.* 2010). Kaeuffer et al. (2007) utilized an empirical dataset from a rigorously studied, isolated subpopulation of wild sheep to confirm that STRUCTURE is not sensitive to the large-scale sources of linkage disequilibrium (mixture and admixture) previously discussed. In contrast, strong background gametic linkage disequilibrium can lead to inflated estimates of *K* when genetic distance values are less than 3 cM (Kaeuffer *et al.* 2007). Because even the presence of a “rare pair” of loci exhibiting strong gametic linkage disequilibrium can bias inference using STRUCTURE, however, researchers should explicitly investigate linkage disequilibrium in their datasets using measures such as *r_LD_*, a between-loci correlation coefficient (Kaeuffer *et al.* 2007). For BAPS, the performance of the linkage model under weak linkage disequilibrium has not been evaluated.

Detecting Weak Structure

The sampling population from which an individual was obtained is informative because, intuitively, it is more likely that the individual belongs to that subpopulation than any other one subpopulation. Therefore, Hubisz et al. (2009) added a fourth ancestry model to STRUCTURE that incorporates known characteristics such as sampling location or phenotypes into population structure inference. This prior population model offers improved ability to detect weak structure, and is also useful for studies with insufficient loci or sample sizes (Hubisz *et al.* 2009). While the prior population model still performs well in cases of strong structure or when the prior information is not informative, it is still recommended to analyze the data under both models for possible biases that may be revealing (Hubisz *et al.* 2009). It should be noted that there are two models in STRUCTURE that incorporate sample origin information: one each for datasets exhibiting weak and strong structure (Hubisz *et al.* 2009). BAPS also contains models that incorporate sampling population information, but in a different fashion. The ‘clustering groups of individuals’ option in BAPS involves splitting and merging the user-specified subpopulations to find the best clustering solution. Compared to the ‘clustering groups of individuals’ option in BAPS, the prior population model in STRUCTURE is more flexible because it allows for the possibility that the subpopulation information does not contribute to clustering inference (Hubisz *et al.* 2009). A second option in BAPS is the ‘trained clustering’ approach, in which unknown individuals are assigned to previously known and defined subpopulations. While appearing to be unnecessary in face of individual-based clustering, the ‘trained clustering’ method is a classification rather than a clustering approach (Manel *et al.* 2005) and is advantageous over individual-based methods when some clusters are small or there is incomplete information on the predefined subpopulations (Corander *et al.* 2006).

Like for fixation indices, recent divergence can obscure population structure to model-based methods and decrease reliability of population structure inference. Thus, Falush et al. (2003) implemented a correlated allele frequency model, an *F* model, to allow structure between recently diverged subpopulations to be detected more easily. This implementation of the *F* model allows for subpopulation-specific magnitudes for drift (Falush *et al.* 2003) and is more robust to unequal sample sizes. Gaggiotti and Foll (2010) applied the same *F* model to fixation indices. Because the *F* model is likely to be useful under various conditions of drift, generations since divergence, and loci used, it is likely prudent to make a robust comparison between the independent and correlated models if recent divergence between given subpopulations is suspected. At low levels of subpopulation differentiation, Waples and Gaggiotti (2006) note it is common for replicate runs of STRUCTURE to not converge, which can be detected from a high variance of posterior probabilities. Excessive variation among replicates can also be caused by violation of method assumptions (Rodríguez-Ramilo & Wang 2012).

Relatedness

Several corollaries to the assumption of Hardy-Weinberg equilibrium have practical implications for model-based inference. First, inbreeding may create a Wahlund effect and a false signature of population structure, thereby leading to an overestimation of admixture or *K* (Falush *et al.* 2003; Gao *et al.* 2007). InStruct was developed to allow more accurate inference of population structure in the presence of inbreeding and also estimate the frequency of selfing (Gao *et al.* 2007). A second corollary is the assumption that individuals are not related by direct descent. Therefore, the presence of such individuals in a dataset may distort Hardy-Weinberg equilibrium and confound parametric population structure inference by, for instance, overestimating *K* (Pritchard *et al.* 2010; Rodríguez-Ramilo & Wang 2012). Anderson and Dunham (2008) reported from empirical and simulated datasets that STRUCTURE depicts false population structure when siblings are present in the data. Rodriquez-Ramilo and Wang (2012) performed a more extensive evaluation of the influence of related individuals on population structure inference using STRUCTURE, InStruct, BAPS, and STRUCTURAMA. Similar to Anderson and Dunham (2008), all programs inferred incorrect population structure in data with related individuals (Rodríguez-Ramilo & Wang 2012). For STRUCTURE, the influence of related individuals on *K* was inconsistent, but inference was more accurate when more subpopulations were present (Rodríguez-Ramilo & Wang 2012) or when differentiation between subpopulations were higher. Both Anderson and Dunham (2008) and Rodriquez-Ramilo and Wang (2012) found that the confounding influence of related individuals is more apparent when the number of loci is increased.

The problem with the presence of related individuals often lies in misinterpreting family structure as population structure, and in addition to STRUCTURE can arise from use of other methods of analysis such as PCA (Anderson & Dunham 2008). When the offending individuals were detected and removed prior to analysis, STRUCTURE was able to correctly infer the true population structure (Anderson & Dunham 2008). The class of methods designed for kinship, parentage, or pedigree-based analyses (Almudevar & Anderson 2012; Jones *et al.* 2010; Wang 2012a; Waples & Waples 2011), including COLONY (Harrison *et al.* 2013; Jones & Wang 2010; Wang 2004) and COANCESTRY (Wang 2011), are recommended to be used to avoid biased inference of population structure and/or in cases of weak population structure (Anderson & Dunham 2008; Palsbøll *et al.* 2010).

Null Alleles

Although designed to handle the ambiguity inherent to dominant genotype data, the recessive allele model in STRUCTURE can be applicable to microsatellites for polyploid organisms in which there is ambiguity in the genotype of heterozygous individuals (Falush *et al.* 2007). However, potential departures from random mating should be carefully considered (Dufresne *et al.* 2014). Additionally, for diploids, alleles or loci that are null with greater frequency may also be analyzed with the recessive allele model. This model is designed for null alleles arising from polymorphism, and not due to experimental errors that should be coded as missing data (Falush *et al.* 2007). Because the other models in STRUCTURE assume loci and/or alleles are missing with uniform frequency, use of the recessive allele model may alleviate bias in situations of unequal rates of null alleles. However, this model should be used with caution if inbreeding is suspected because estimates of null alleles may be artificially inflated (Falush *et al.* 2007).

Appendix S5: Model-based K inference

Ad-Hoc Methods

Identifying the number of subpopulations of an organism is a central problem in population genetic inference. Conceptual difficulties arise because descriptions and definitions of a ‘population’ of organisms can vary widely based on perspective (ecological versus evolutionary), system, or the questions being addressed, and these concepts may not correlate with genetic clustering results (Waples & Gaggiotti 2006). Moreover, similar to the exploratory methods discussed above, and aside from problems from population models discussed above, there are procedural and statistical difficulties in estimating *K* using model-based methods. This problem is clearly evident with STRUCTURE. Because each STRUCTURE run requires *K* to be fixed *a priori*, *K* cannot be formally estimated. Instead, *ad hoc* methods have been proposed that rely on the posterior probabilities of STRUCTURE runs. Pritchard et al. (2000) proposed selecting the *K* value that produced the highest posterior probabilities. When *K* is increased, selecting the value at which probabilities plateau has been proposed (Rosenberg *et al.* 2002) and reported to work well for STRUCTURE, and particularly well for TESS and GENECLUST (Chen *et al.* 2007). More formally, Evanno et al. (2005) suggested the Δ*K* method that evaluates the rate of change in probabilities as the value of *K* is increased. However, the Evanno et al. (2005) method has been reported to be not different from (Waples & Gaggiotti 2006) or inferior to (Duchesne & Turgeon 2012) the original method proposed by Pritchard et al. (2000). The non-model based iterated reallocation method found in FLOCK has been proposed as superior to methods associated with STRUCTURE (Duchesne & Turgeon 2012). Moreover, especially for haploid or highly selfing organisms, the two methods (Evanno *et al.* 2005; Pritchard *et al.* 2000) may be unable to identify the number of clusters when the number of subpopulations is large (Fogelqvist *et al.* 2010). Finally, obtaining increasing likelihood values when *K* is increased could indicate the data is being over-fit rather than revealing true structure (Lee *et al.* 2009). The median probability and the change in median probability of replicate runs may also be evaluated for selecting *K* to avoid bias from outlier runs (Saisho & Purugganan 2007). Another method for selecting *K* is the deviance information criterion (DIC), which is available in TESS and evaluated similarly to other methods by plotting against *K* values (François & Durand 2010). As implemented in InStruct, DIC has been shown to outperform most *K* selection methods including Δ*K*, STRUCTURAMA, BAPS, and PCA under various demographic scenarios such as high migration (Gao *et al.* 2011a).

The Evanno et al. (2005) method has been made more accessible by its implementation into the program STRUCTURE HARVESTER, which also offers convenient summarization and plotting of trace data from many runs (Earl & VonHoldt 2011). Similarly, the program CorrSieve (Campana *et al.* 2011) collates and summarizes STRUCTURE output and includes the Evanno et al. (2005) method, but contains additional functionality. CorrSieve uses STRUCTURE’s output to calculate Δ*F_ST_*, which can be used to supplement the Δ*K* of Evanno et al. (Evanno *et al.* 2005), and implements correlation analysis of the ancestry coefficients to provide evidence for the most stable *K* value across replicate runs (Campana *et al.* 2011).

BAPS implements several methods to estimate *K*. Like STRUCTURE, BAPS can perform clustering based on user-specified *K* values (Corander *et al.* 2008a). However, BAPS also implements methods to evaluate all values of *K* from *K* = 1 to a user-specified maximum. Thirdly, BAPS can determine the probability of various arrangements of subpopulations or individuals specified by the researcher. These options for determining *K* are applicable to any of the clustering models in BAPS. GENELAND also evaluates *K* values up to a maximum, but does so without user specification and returns posterior probabilities of *K* (François & Durand 2010; Guillot *et al.* 2005). When choosing *K*, researchers should not rely on direct comparisons of the optimal *K* value selected by different methods because most methods differ in their assumptions (François & Durand 2010). Instead, formal methods to select an accurate model are advised (see François & Durand 2010 for discussion).

Formal Inference

Pella and Masuda (2006) used a Dirichlet process prior to address the *K* selection problem. Also known as the ‘Chinese Restaurant Table Process,’ the Dirichlet process treats *K* as a random variable and allows it to be simultaneously estimated along with the assignment of individuals to subpopulations. This implementation is available in the program HWLER (Pella & Masuda 2006). The Dirichlet process was also implemented in the program STRUCTURAMA, which did not include admixture, but contains improved methods to summarize Bayesian clustering analyses (Huelsenbeck & Andolfatto 2007). However, the chain mixing method employed by HWLER is faster than that used by STRUCTURAMA (Onogi *et al.* 2011). In a comparison to STRUCTURE, Hausdorf and Hennig (2010) evaluated the performance of STRUCTURAMA at detecting species and assigning individuals to species among two empirical microsatellite datasets and found STRUCTURAMA to be superior in both cases. As an improvement, Huelsenbeck et al. (2011) added Dirichlet process model to STRUCTURAMA that includes admixture. Concurrently, Shringarpure et al. (2011) released StructHDP, which incorporates an admixture model with the Dirichlet process prior for inferring *K*.

Caution should be used with setting the prior on allele frequencies with any Dirichlet process method because it can significantly influence accuracy. However, users cannot specify this prior in STRUCTURAMA, and it is not clear how the prior is implemented in this program (see Onogi *et al.* 2011 for discussion). Onogi et al. (2011) developed a third implementation of the Dirichlet process in the program DPART, which includes the more efficient sampler found in HWLER and the ability to modify the allele frequency prior, but does not include an admixture model. Another advantage of the Dirichlet process method is its superior performance compared to methods such as STRUCTURE when sample sizes from different subpopulations are unequal and when differentiation between subpopulations is low (Onogi *et al.* 2011). This deficiency in STRUCTURE was most apparent for the correlated allele frequency model (Onogi *et al.* 2011). In contrast, STRUCTURE’s correlated allele frequency model can outperform STRUCTURAMA at inferring *K* under certain demographic scenarios such as high migration and larger *K* values (Gao *et al.* 2011b).

Appendix S6: Summary of use of descriptive statistics for inferring migration

The disruption of allele fixation in a given subpopulation by migration is detectable by descriptive statistics, which are indirect migration inference methods. Thus, *F_ST_* naturally includes migration (*m*) as a component, and is widely used to infer patterns of migration due to the commonly cited simple, inverse relationship between these parameters [given by *F_ST_* = 1 / (4*N_e_m* + 1) ]. However, as discussed above for population structure, this relationship is valid only in the infinite island model that has reached drift-migration equilibrium (Holsinger & Weir 2009; Lowe & Allendorf 2010; Meirmans & Hedrick 2011; Whitlock & McCauley 1999). In practice, subpopulation sizes or migration rates are likely to be unequal among subpopulations. Moreover, microsatellites are typically employed to study populations in non-equilibrium conditions (Whitlock & McCauley 1999), which can lead to overestimated migration (Lowe & Allendorf 2010). Finally, the above relationship is derived from a version that includes mutation, thus the simplified version is valid only if the mutation rate is much lower than the migration rate. When mutation approaches or exceeds migration, the mutation term cannot be ignored and confounds inference of migration using *F­_ST_* for highly polymorphic microsatellite loci (Hardy *et al.* 2003).

The influence of migration on population structure can be overestimated using *F_ST_* when there are too few subpopulations or for high migration rates (Song *et al.* 2006). Imposing constraints on allele size can lead to an overestimation of migration (Gaggiotti *et al.* 1999). Rousset (1996) reported that *R_ST_* has the same relationship with migration as *F_ST_* when mutation and migration are rare, but Song et al. (Song *et al.* 2011) showed using exact moment calculations that *R_ST_* is a good measure of migration, even at high mutation rates or when mutations deviate from the SMM. Estimators of *F_ST_* are more reliable for inferring migration when population sizes, sample sizes, or the number of loci are small, but *R_ST_* estimators perform better than *F_ST_* when these values are large (Gaggiotti *et al.* 1999). Although estimates of *F_ST_* may be reliable under certain conditions to infer how migration has shaped population structure (Cockerham & Weir 1993), *F_ST_* is a nonlinear function of *N_e_m* and thus the error inherent in estimating *F_ST_* is amplified when used to estimate values of migration (Whitlock & McCauley 1999). *G_ST_* is often cited as a good tool to infer migration (Cockerham & Weir 1993; Jost 2009; Ryman & Leimar 2009), but only under certain conditions, and as an analogue of *F_ST_* it is likely influenced by the same factors as *F_ST_* above (Leng & Zhang 2013). The performance of *F_ST_* analogues for estimation of migration has not been investigated in detail.

While *D* is influenced by migration, this relationship is not straightforward and *D* should not be used to estimate migration (Jost 2009; Kronholm *et al.* 2010; Whitlock 2011). However, *D* may be able to ascertain the order of magnitude of migration or determine if a locus is more influenced by mutation or migration (Whitlock 2011). In contrast to *D*, Sherwin et al. (2006) proposed that entropy-based methods can be used to estimate migration. Using an empirically derived equation relating MI with effective population size and migration rate from simulations, migration rates could be estimated from several data sets, including an experimental population of *Drosophila* (Sherwin *et al.* 2006)*.* This method performed well compared to *F_ST_* even at high migration rates, for small population sizes, and at a high rate of mutation (10^-2^) (Sherwin *et al.* 2006). Despite these results, however, entropy-based methods have only been employed to estimate migration in select studies (Karlin *et al.* 2011; Rossetto *et al.* 2011) and their use requires further investigation. For example, it is unclear if these methods can accurately estimate migration when factors other than migration are responsible for low levels of population structure.

Masucci et al. (2011) have recently proposed a more theoretical approach to using entropy to infer migration. This method uses the Jensen-Shannon divergence, which explicitly quantifies the information flow between two groups. The connectivity of subpopulations within the network is inferred using a threshold. Masucci et al. (2011) applied this method to a microsatellite dataset of *Posidonia oceanica*, a diploid seagrass, and successfully recovered networks and degrees of connectivity that conformed to previous studies of this system. Jensen-Shannon divergence has promise for migration inference because it allows unequal population sizes, accounts for correlations among loci, and also infers a direction of flow, but it has not yet been studied or made accessible. Other indirect methods include genetic distances (Dyer *et al.* 2010; Jaquiéry *et al.* 2011), and rare alleles or allele covariance (Broquet *et al.* 2009; Lowe & Allendorf 2010; Waples & Gaggiotti 2006).

Appendix S7: Overview of methods for ancestral inference

Coalescent Estimation

Coalescent methods sample and analyze genealogies back in time to the common ancestor of a given sample. The program MIGRATE uses the coalescent to estimate subpopulation-specific Θ and bi-directional migration rates in a likelihood framework for two subpopulations (Beerli & Felsenstein 1999) or pair-wise among any number of subpopulations (Beerli & Felsenstein 2001). This method assumes ancient divergence and constant *N_e_*. MIGRATE was later upgraded with the option for Bayesian estimation, some user-specified population models such as the stepping stone model, and tests for panmixia or model selection (Beerli & Palczewski 2010). A new method, which models migrations as probabilities rather than discrete events, offers improved performance in cases of high migration but has not yet been incorporated into the MIGRATE program (Palczewski & Beerli 2013). LAMARC 2.0 is a compilation of several previous programs and has similar methods as earlier versions of MIGRATE, such as estimation of Θ and pair-wise migration rates (Kuhner 2006). Unlike MIGRATE, LAMARC 2.0 can infer the rate of exponential growth for each subpopulation.

As discussed for population structure inference, correctly identifying population structure when two isolated subpopulations have recently diverged is a significant problem in population genetics. However, many methods in population genetics cannot distinguish this case of demographic history from that of two subpopulations that diverged a long time ago but are connected by migration (Nielsen & Wakeley 2001). Therefore, Nielsen and Wakeley (2001) developed the ‘isolation with migration’ (IM) model to allow simultaneous estimation of bi-directional migration rates, divergence times, and Θ for two contemporary subpopulations using an MCMC algorithm. The IM programs have the ability to analyze multiple loci (Hey & Nielsen 2004), microsatellite data via the SMM (Hey *et al.* 2004), and more than two contemporary subpopulations (Hey 2010). The IM model has recently been extended to allow the assignment of individuals to subpopulations (Choi & Hey 2011) and the detection of loci under selection (Sousa *et al.* 2013), but these capabilities do not appear to be included in the most recent version of IMa2.

Bayesian Evolutionary Analysis by Sampling Trees (BEAST) is a package of methods for inference of gene or species phylogenies (Bouckaert *et al.* 2013; Drummond *et al.* 2012). Although originally developed for and widely applied to analysis of species or higher taxonomic levels, BEAST estimates divergence times and population sizes using the coalescent and can be used for typical population level divergence times under certain circumstances, such as no gene flow (Heled & Drummond 2010) or when migration is not too high (Heled *et al.* 2013). BEAST has recently been extended to allow analysis of microsatellite data (Wu & Drummond 2011) and has the potential to be a powerful tool in population genetic inference because it allows flexible, locus-specific model specification for different marker types and multilocus estimation of ‘species’ trees (Heled & Drummond 2010). While Heled et al. (2013) showed that the new implementation of BEAST (Heled & Drummond 2010) can distinguish two populations that have recently diverged and are exchanging migrants, use of BEAST in these situations and/or with microsatellites have not been thoroughly investigated (Heled *et al.* 2013).

Coalescent-based methods are especially powerful tools for population genetic inference, but this power comes at the cost of several practical limitations (Kuhner 2009; Pinho & Hey 2010). Achieving and confirming convergence of program runs is a significant overall challenge (Hey & Nielsen 2004). To combat this problem, these programs employ Metropolis coupling, or multiple, simultaneous ‘heated’ chains, that allow more thorough searching (Hey & Nielsen 2004). Many chains are required, such as tens or in excess of 100 for IM programs, and individual analyses typically need to run for a long time to achieve convergence (Hey 2010; Pinho & Hey 2010). In general, convergence success and run times are proportional to model complexity (e.g., the number of subpopulations) and inversely proportional to information content of the dataset (Hey 2011; Kuhner 2009).

Some of these methods are also used to analyze DNA sequence data in the related field of phylogeography (reviewed in Brito & Edwards 2009; Chan *et al.* 2011; Garrick *et al.* 2010; Knowles 2009; Nielsen & Beaumont 2009).

Approximate Bayesian Computation

When applied to population genetics, approximate Bayesian computation (ABC) attempts to answer the following question: which model(s) of evolutionary history could give rise to the summary statistics calculated from the sample dataset at hand? Briefly, many datasets are simulated under various hypothesized demographic scenarios, and summary statistics calculated from these simulations are compared to the actual sample to determine which hypothesized scenario best approximates the observed empirical data. A major advantage of ABC is that because it does not involve explicit calculations of likelihood functions, a large variety of complex demographic scenarios that are inaccessible to other methods can be analyzed (Beaumont 2010; Bertorelle *et al.* 2010; Csilléry *et al.* 2010).

Since becoming more accessible in programs such as DIYABC (Cornuet *et al.* 2010) and the R package abc (Csilléry *et al.* 2012), ABC is being actively applied in population genetic studies, with microsatellites as the most popular marker (Bertorelle *et al.* 2010). However, ABC is not free of significant time requirements as it requires careful choice of summary statistics, and model fitting and checking steps (Aeschbacher *et al.* 2012; Bertorelle *et al.* 2010; Csilléry *et al.* 2010; De Mita & Siol 2012; Peter *et al.* 2010; Sousa *et al.* 2012; Sousa *et al.* 2009; Sunnåker *et al.* 2013), especially for scenarios investigating a limited number of parameters, such as migration, among multiple populations (Aeschbacher *et al.* 2013). The R package EasyABC incorporates sequential and MCMC sampling schemes to greatly improve efficiency of these steps over the standard rejection schemes and allows easy integration with the previously developed abc package (Jabot *et al.* 2013). These and other considerations for the practical applications of ABC have been discussed (Estoup *et al.* 2012; Robert *et al.* 2011) and thoroughly reviewed (Bertorelle *et al.* 2010; Csilléry *et al.* 2010; Sunnåker *et al.* 2013).

References

Aeschbacher S, Beaumont MA, Futschik A (2012) A novel approach for choosing summary statistics in approximate Bayesian computation. *Genetics* **192**, 1027-1047.

Aeschbacher S, Futschik A, Beaumont MA (2013) Approximate Bayesian computation for modular inference problems with many parameters: the example of migration rates. *Molecular Ecology* **22**, 987-1002.

Albatineh AN, Niewiadomska-Bugaj M (2011) MCS: A method for finding the number of clusters. *Journal of Classification* **28**, 184-209.

Almudevar A, Anderson EC (2012) A new version of PRT software for sibling groups reconstruction with comments regarding several issues in the sibling reconstruction problem. *Molecular Ecology Resources* **12**, 164-178.

Anderson CD, Epperson BK, Fortin M-J, Holderegger R, James PMA, Rosenberg MS, Scribner KT, Spear S (2010) Considering spatial and temporal scale in landscape-genetic studies of gene flow. *Molecular Ecology* **19**, 3565-3575.

Anderson EC, Dunham KK (2008) The influence of family groups on inferences made with the program Structure. *Molecular Ecology Resources* **8**, 1219-1229.

Anderson LL, Hu FS, Paige KN (2011) Phylogeographic history of white spruce during the last glacial maximum: uncovering cryptic refugia. *Journal of Heredity* **102**, 207-216.

Andrew RL, Ostevik KL, Ebert DP, Rieseberg LH (2012) Adaptation with gene flow across the landscape in a dune sunflower. *Molecular Ecology* **21**, 2078-2091.

Atallah ZK, Maruthachalam K, du Toit L, Koike ST, Michael Davis R, Klosterman SJ, Hayes RJ, Subbarao KV (2010) Population analyses of the vascular plant pathogen *Verticillium dahliae* detect recombination and transcontinental gene flow. *Fungal Genetics and Biology* **47**, 416-422.

Balding DJ (2003) Likelihood-based inference for genetic correlation coefficients. *Theoretical Population Biology* **63**, 221-230.

Balloux F, Goudet J (2002) Statistical properties of population differentiation estimators under stepwise mutation in a finite island model. *Molecular Ecology* **11**, 771-783.

Balloux F, Lugon-Moulin N (2002) The estimation of population differentiation with microsatellite markers. *Molecular Ecology* **11**, 155-165.

Beaumont MA (2005) Adaptation and speciation: what can *F(ST)* tell us? *Trends in Ecology & Evolution* **20**, 435-440.

Beaumont MA (2010) Approximate Bayesian computation in evolution and ecology. *Annual Review of Ecology, Evolution, and Systematics* **41**, 379-406.

Beerli P, Felsenstein J (1999) Maximum-likelihood estimation of migration rates and effective population numbers in two populations using a coalescent approach. *Genetics* **152**, 763-773.

Beerli P, Felsenstein J (2001) Maximum likelihood estimation of a migration matrix and effective population sizes in *n* subpopulations by using a coalescent approach. *Proceedings of the National Academy of Sciences* **98**, 4563-4568.

Beerli P, Palczewski M (2010) Unified framework to evaluate panmixia and migration direction among multiple sampling locations. *Genetics* **185**, 313-326.

Bertorelle G, Benazzo A, Mona S (2010) ABC as a flexible framework to estimate demography over space and time: some cons, many pros. *Molecular Ecology* **19**, 2609-2625.

Bird SC (2012) Towards improvements in the estimation of the coalescent: implications for the most effective use of Y chromosome short tandem repeat mutation rates. *PLoS One* **7**, e48638.

Blair C, Weigel DE, Balazik M, Keeley ATH, Walker FM, Landguth E, Cushman S, Murphy M, Waits L, Balkenhol N (2012) A simulation-based evaluation of methods for inferring linear barriers to gene flow. *Molecular Ecology Resources* **12**, 822-833.

Blum MJ, Bagley MJ, Walters DM, Jackson SA, Daniel FB, Chaloud DJ, Cade BS (2012) Genetic diversity and species diversity of stream fishes covary across a land-use gradient. *Oecologia* **168**, 83-95.

Bohling JH, Adams JR, Waits LP (2013) Evaluating the ability of Bayesian clustering methods to detect hybridization and introgression using an empirical red wolf data set. *Molecular Ecology* **22**, 74-86.

Bouaziz M, Paccard C, Guedj M, Ambroise C (2012) SHIPS: Spectral Hierarchical Clustering for the Inference of Population Structure in genetic gtudies. *PLoS One* **7**, e45685.

Bouckaert R, Heled J, Kühnert D, Vaughan T, Wu C-H, Xie D, Suchard M, Rambaut A, Drummond A (2013) BEAST2: A software platform for Bayesian evolutionary analysis. *available at http://beast2.org/*.

Bowcock AM, Ruiz-Linares A, Tomfohrde J, Minch E, Kidd JR, Cavalli-Sforza LL (1994) High resolution of human evolutionary trees with polymorphic microsatellites. *Nature* **368**, 455-457.

Brito PH, Edwards SV (2009) Multilocus phylogeography and phylogenetics using sequence-based markers. *Genetica* **135**, 439-455.

Brock G, Pihur V, Datta S, Datta S (2008) clValid : An R Package for cluster validation. *Journal Of Statistical Software* **25**, 1-22.

Broquet T, Yearsley J, Hirzel AH, Goudet J, Perrin N (2009) Inferring recent migration rates from individual genotypes. *Molecular Ecology* **18**, 1048-1060.

Calinski T, Harabasz J (1974) A dendrite method for cluster analysis. *Communications in Statistics - Theory and Methods* **3**, 1-27.

Campana MG, Hunt HV, Jones H, White J (2011) *CorrSieve*: software for summarizing and evaluating Structure output. *Molecular Ecology Resources* **11**, 349-352.

Cavalli-Sforza LL (1966) Population structure and human evolution. *Proceedings of the Royal Society B: Biological Sciences* **164**, 362-379.

Chakraborty R, Nei M (1982) Genetic differentiation of quantitative characters between populations or species I. Mutation and randome genetic drift. *Genetical Research* **39**, 303-314.

Chan LM, Brown JL, Yoder AD (2011) Integrating statistical genetic and geospatial methods brings new power to phylogeography. *Molecular Phylogenetics and Evolution* **59**, 523-537.

Chen C, Durand E, Forbes F, François O (2007) Bayesian clustering algorithms ascertaining spatial population structure: a new computer program and a comparison study. *Molecular Ecology Notes* **7**, 747-756.

Choi SC, Hey J (2011) Joint inference of population assignment and demographic history. *Genetics* **189**, 561-577.

Cockerham CC, Weir BS (1993) Estimation of gene flow from *F*-statistics. *Evolution* **47**, 855-863.

Corander J, Marttinen P (2006) Bayesian identification of admixture events using multilocus molecular markers. *Molecular Ecology* **15**, 2833-2843.

Corander J, Marttinen P, Mäntyniemi S (2006) A Bayesian method for identification of stock mixtures from molecular marker data. *Fishery Bulletin* **104**, 550-558.

Corander J, Marttinen P, Sirén J, Tang J (2008a) Enhanced Bayesian modelling in BAPS software for learning genetic structures of populations. *BMC Bioinformatics* **9**, 539.

Corander J, Sirén J, Arjas E (2008b) Bayesian spatial modeling of genetic population structure. *Computational Statistics* **23**, 111-129.

Corander J, Tang J (2007) Bayesian analysis of population structure based on linked molecular information. *Mathematical Biosciences* **205**, 19-31.

Corander J, Waldmann P, Marttinen P, Sillanpää MJ (2004) BAPS 2: enhanced possibilities for the analysis of genetic population structure. *Bioinformatics* **20**, 2363-2369.

Corander J, Waldmann P, Sillanpää MJ (2003) Bayesian analysis of genetic differentiation between populations. *Genetics* **163**, 367-374.

Cornuet J-M, Ravigné V, Estoup A (2010) Inference on population history and model checking using DNA sequence and microsatellite data with the software DIYABC (v1.0). *BMC Bioinformatics* **11**, 401.

Csilléry K, Blum MGB, Gaggiotti OE, François O (2010) Approximate Bayesian Computation (ABC) in practice. *Trends in Ecology & Evolution* **25**, 410-418.

Csilléry K, François O, Blum MGB (2012) abc: an R package for approximate Bayesian computation (ABC). *Methods in Ecology and Evolution* **3**, 475-479.

De Mita S, Siol M (2012) EggLib: processing, analysis and simulation tools for population genetics and genomics. *BMC Genetics* **13**, 27.

DeGiorgio M, Rosenberg NA (2013) Geographic sampling scheme as a determinant of the major axis of genetic variation in principal components analysis. *Molecular Biology and Evolution* **30**, 480-488.

Dewar RC, Sherwin WB, Thomas E, Holleley CE, Nichols RA (2011) Predictions of single-nucleotide polymorphism differentiation between two populations in terms of mutual information. *Molecular Ecology* **20**, 3156-3166.

Drummond AJ, Suchard MA, Xie D, Rambaut A (2012) Bayesian phylogenetics with BEAUti and the BEAST 1.7. *Molecular Biology and Evolution* **29**, 1969-1973.

Duchesne P, Turgeon J (2009) FLOCK: a method for quick mapping of admixture without source samples. *Molecular Ecology Resources* **9**, 1333-1344.

Duchesne P, Turgeon J (2012) FLOCK provides reliable solutions to the "number of populations" problem. *The Journal of Heredity* **103**, 734-743.

Dufresne F, Marková S, Vergilino R, Ventura M, Kotlík P (2011) Diversity in the reproductive modes of European *Daphnia pulicaria* deviates from the geographical parthenogenesis. *PLoS One* **6**, e20049.

Dufresne F, Stift M, Vergilino R, Mable BK (2014) Recent progress and challenges in population genetics of polyploid organisms: an overview of current state-of-the-art molecular and statistical tools. *Molecular Ecology* **23**, 40-69.

Durand E, Chen C, François O (2009a) Comment on 'On the inference of spatial structure from population genetics data'. *Bioinformatics* **25**, 1802-1804.

Durand E, Jay F, Gaggiotti OE, François O (2009b) Spatial inference of admixture proportions and secondary contact zones. *Molecular Biology and Evolution* **26**, 1963-1973.

Dyer RJ (2009) GeneticStudio: a suite of programs for spatial analysis of genetic-marker data. *Molecular Ecology Resources* **9**, 110-113.

Dyer RJ, Nason JD (2004) Population Graphs: the graph theoretic shape of genetic structure. *Molecular Ecology* **13**, 1713-1727.

Dyer RJ, Nason JD, Garrick RC (2010) Landscape modelling of gene flow: improved power using conditional genetic distance derived from the topology of population networks. *Molecular Ecology* **19**, 3746-3759.

Earl DA, VonHoldt BM (2011) STRUCTURE HARVESTER: a website and program for visualizing STRUCTURE output and implementing the Evanno method. *Conservation Genetics Resources* **4**, 359-361.

Edelaar P, Björklund M (2011) If *F(ST)* does not measure neutral genetic differentiation, then comparing it with *Q(ST)* is misleading. Or is it? *Molecular Ecology* **20**, 1805-1812.

Edelaar P, Burraco P, Gomez-Mestre I (2011) Comparisons between *Q(ST)* and *F(ST)*—how wrong have we been? *Molecular Ecology* **20**, 4830-4839.

Estoup A, Angers B (1998) Microsatellites and minisatellites for molecular ecology: theoretical and empirical considerations. In: *Advances in Molecular Ecology* (ed. Carvalho GR), pp. 55-86. IOS Press, Amsterdam.

Estoup A, Lombaert E, Marin J-M, Guillemaud T, Pudlo P, Robert CP, Cornuet J-M (2012) Estimation of demo-genetic model probabilities with Approximate Bayesian Computation using linear discriminant analysis on summary statistics. *Molecular Ecology Resources* **12**, 846-855.

Evanno G, Regnaut S, Goudet J (2005) Detecting the number of clusters of individuals using the software STRUCTURE: a simulation study. *Molecular Ecology* **14**, 2611-2620.

Excoffier L, Smouse PE, Quattro JM (1992) Analysis of molecular variance inferred from metric distances among DNA haplotypes: application to human mitrochondrial DNA restriction data. *Genetics* **131**, 479-491.

Falush D, Stephens M, Pritchard JK (2003) Inference of population structure using multilocus genotype data: linked loci and correlated allele frequencies. *Genetics* **164**, 1567-1587.

Falush D, Stephens M, Pritchard JK (2007) Inference of population structure using multilocus genotype data: dominant markers and null alleles. *Molecular Ecology Notes* **7**, 574-578.

Felsenstein J (2004) *Inferring Phylogenies* Sinauer Associates, Sunderland, MA.

Filippone M, Camastra F, Masulli F, Rovetta S (2008) A survey of kernel and spectral methods for clustering. *Pattern Recognition* **41**, 176-190.

Fogelqvist J, Niittyvuopio A, Ågren J, Savolainen O, Lascoux M (2010) Cryptic population genetic structure: the number of inferred clusters depends on sample size. *Molecular Ecology Resources* **10**, 314-323.

Fraley C, Raftery AE (1998) How many clusters? Which clustering method? Answers via model-based cluster analysis. *The Computer Journal* **41**, 578-588.

Fraley C, Raftery AE (2003) Enhanced model-based clustering, density estimation, and discriminant analysis software: MCLUST. *Journal of Classification* **20**, 263-286.

François O, Currat M, Ray N, Han E, Excoffier L, Novembre J (2010) Principal component analysis under population genetic models of range expansion and admixture. *Molecular Biology and Evolution* **27**, 1257-1268.

François O, Durand E (2010) Spatially explicit Bayesian clustering models in population genetics. *Molecular Ecology Resources* **10**, 773-784.

Fu R, Dey DK, Holsinger KE (2005) Bayesian models for the analysis of genetic structure when populations are correlated. *Bioinformatics* **21**, 1516-1529.

Fu R, E. Gelfand A, Holsinger KE (2003) Exact moment calculations for genetic models with migration, mutation, and drift. *Theoretical Population Biology* **63**, 231-243.

Gaggiotti OE, Foll M (2010) Quantifying population structure using the *F*-model. *Molecular Ecology Resources* **10**, 821-830.

Gaggiotti OE, Lange O, Rassmann K, Gliddon C (1999) A comparison of two indirect methods for estimating average levels of gene flow using microsatellite data. *Molecular Ecology* **8**, 1513-1520.

Galluccio L, Michel O, Comon P, Hero AO (2012) Graph based k-means clustering. *Signal Processing* **92**, 1984-1970.

Gao H, Bryc K, Bustamante CD (2011a) On identifying the optimal number of population clusters via the deviance information criterion. *PLoS One* **6**, e21014.

Gao H, Williamson S, Bustamante CD (2007) A Markov chain Monte Carlo approach for joint inference of population structure and inbreeding rates from multilocus genotype data. *Genetics* **176**, 1635-1651.

Gao S, Sung W-K, Nagarajan N (2011b) Opera: reconstructing optimal genomic scaffolds with high-throughput paired-end sequences. *Journal of Computational Biology* **18**, 1681-1691.

Gao X, Martin R (2009) Using allele sharing distance for detecting human population stratification. *Human Heredity* **68**, 182-191.

Gao X, Starmer JD (2008) AWclust: point-and-click software for non-parametric population structure analysis. *BMC Bioinformatics* **9**, 77.

Garrick RC, Caccone A, Sunnucks P (2010) Inference of population history by coupling exploratory and model-driven phylogeographic analyses. *International Journal of Molecular Sciences* **11**, 1190-1227.

Gerlach G, Jueterbock A, Kraemer P, Deppermann J, Harmand P (2010) Calculations of population differentiation based on *G(ST)* and *D*: forget *G(ST)* but not all of statistics! *Molecular Ecology* **19**, 3845-3852.

Gillet EM (2013) DifferInt : compositional differentiation among populations at three levels of genetic integration. *Molecular Ecology Resources* **13**, 953-964.

Gompert Z, Buerkle CA (2013) Analyses of genetic ancestry enable key insights for molecular ecology. *Molecular Ecology* **22**, 5278-5294.

Goodman SJ (1997) *R(ST)* Calc: a collection of computer programs for calculating estimates of genetic differentiation from microsatellite data and determining their significance. *Molecular Ecology* **6**, 881-885.

Gopal V, Fuentes C, Casella G (2012) bayesclust: An R package for testing and searching for significant clusters. *Journal Of Statistical Software* **47**, 1-21.

Goss EM, Larsen M, Chastagner GA, Givens DR, Grünwald NJ (2009) Population genetic analysis infers migration pathways of *Phytophthora ramorum* in US nurseries. *PLoS Pathogens* **5**, e1000583.

Gregorius H-R (2010) Linking diversity and differentiation. *Diversity* **2**, 370-394.

Gregorius H-R, Degen B, König A (2007) Problems in the analysis of genetic differentiation among populations – a case study in *Quercus robur*. *Silvae Genetica* **56**, 190-199.

Guillot G (2008) Inference of structure in subdivided populations at low levels of genetic differentiation—the correlated allele frequencies model revisited. *Bioinformatics* **24**, 2222-2228.

Guillot G (2009a) On the inference of spatial structure from population genetics data. *Bioinformatics* **25**, 1796-1801.

Guillot G (2009b) Response to comment on 'On the inference of spatial structure from population genetics data'. *Bioinformatics* **25**, 1805-1806.

Guillot G, Leblois R, Coulon A, Frantz AC (2009) Statistical methods in spatial genetics. *Molecular Ecology* **18**, 4734-4756.

Guillot G, Mortier F, Estoup A (2005) GENELAND: a computer package for landscape genetics. *Molecular Ecology Notes* **5**, 712-715.

Guillot G, Renaud S, Ledevin R, Michaux J, Claude J (2012) A unifying model for the analysis of phenotypic, genetic, and geographic data. *Systematic Biology* **61**, 897-911.

Guillot G, Santos F (2009) A computer program to simulate multilocus genotype data with spatially autocorrelated allele frequencies. *Molecular Ecology Resources* **9**, 1112-1120.

Guillot G, Santos F, Estoup A (2008) Analysing georeferenced population genetics data with Geneland: a new algorithm to deal with null alleles and a friendly graphical user interface. *Bioinformatics* **24**, 1406-1407.

Haasl RJ, Payseur BA (2010) The number of alleles at a microsatellite defines the allele frequency spectrum and facilitates fast accurate estimation of theta. *Molecular Biology and Evolution* **27**, 2702-2715.

Handl J, Knowles J, Kell DB (2005) Computational cluster validation in post-genomic data analysis. *Bioinformatics* **21**, 3201-3212.

Hardy OJ, Charbonnel N, Fréville H, Heuertz M (2003) Microsatellite allele sizes: a simple test to assess their significance on genetic differentiation. *Genetics* **163**, 1467-1482.

Harris P, Brunsdon C, Charlton M (2011) Geographically weighted principal components analysis. *International Journal of Geographical Information Science* **25**, 1717-1736.

Harrison HB, Saenz-Agudelo P, Planes S, Jones GP, Berumen ML (2013) Relative accuracy of three common methods of parentage analysis in natural populations. *Molecular Ecology* **22**, 1158-1170.

Hausdorf B, Hennig C (2010) Species delimitation using dominant and codominant multilocus markers. *Systematic Biology* **59**, 491-503.

Hedrick PW (2005) A standardized genetic differentiation measure. *Evolution* **59**, 1633-1638.

Heled J, Bryant D, Drummond AJ (2013) Simulating gene trees under the multispecies coalescent and time-dependent migration. *BMC Evolutionary Biology* **13**, 44.

Heled J, Drummond AJ (2010) Bayesian inference of species trees from multilocus data. *Molecular Biology and Evolution* **27**, 570-580.

Heller R, Siegismund HR (2009) Relationship between three measures of genetic differentiation *G(ST)*, *D(EST)* and *G'(ST)*: how wrong have we been? *Molecular Ecology* **18**, 2080-2083.

Hey J (2010) Isolation with migration models for more than two populations. *Molecular Biology and Evolution* **27**, 905-920.

Hey J (2011) *Documentation for IMa2* Department of Genetics, Rutgers University, New Brunswick, NJ.

Hey J, Nielsen R (2004) Multilocus methods for estimating population sizes, migration rates and divergence time, with applications to the divergence of *Drosophila pseudoobscura* and *D. persimilis*. *Genetics* **167**, 747-760.

Hey J, Won Y-J, Sivasundar A, Nielsen R, Markert JA (2004) Using nuclear haplotypes with microsatellites to study gene flow between recently separated Cichlid species. *Molecular Ecology* **13**, 909-919.

Holderegger R, Wagner HH (2008) Landscape genetics. *BioScience* **58**, 199-207.

Holsinger KE, Weir BS (2009) Genetics in geographically structured populations: defining, estimating and interpreting *F(ST)*. *Nature Reviews Genetics* **10**, 639-650.

Hubisz MJ, Falush D, Stephens M, Pritchard JK (2009) Inferring weak population structure with the assistance of sample group information. *Molecular Ecology Resources* **9**, 1322-1332.

Huelsenbeck JP, Andolfatto P (2007) Inference of population structure under a Dirichlet process model. *Genetics* **175**, 1787-1802.

Huelsenbeck JP, Andolfatto P, Huelsenbeck ET (2011) Structurama: Bayesian inference of population structure. *Evolutionary Bioinformatics* **7**, 55-59.

Intarapanich A, Shaw PJ, Assawamakin A, Wangkumhang P, Ngamphiw C, Chaichoompu K, Piriyapongsa J, Tongsima S (2009) Iterative pruning PCA improves resolution of highly structured populations. *BMC Bioinformatics* **10**, 382.

Jabot F, Faure T, Dumoulin N (2013) EasyABC: performing efficient approximate Bayesian computation sampling schemes using R. *Methods in Ecology and Evolution* **4**, 684-687.

Jain AK, Murty MN, Flynn PJ (1999) Data clustering: a review. *ACM Computing Surveys* **31**, 264-323.

Jakobsson M, Edge MD, Rosenberg NA (2013) The relationship between *F(ST)* and the frequency of the most frequent allele. *Genetics* **193**, 515-528.

Jaquiéry J, Broquet T, Hirzel AH, Yearsley J, Perrin N (2011) Inferring landscape effects on dispersal from genetic distances: how far can we go? *Molecular Ecology* **20**, 692-705.

Jay JJ, Eblen JD, Zhang Y, Benson M, Perkins AD, Saxton AM, Voy BH, Chesler EJ, Langston MA (2012) A systematic comparison of genome-scale clustering algorithms. *BMC Bioinformatics* **13**, S7.

Jombart T (2008) adegenet: a R package for the multivariate analysis of genetic markers. *Bioinformatics* **24**, 1403-1405.

Jombart T, Devillard S, Balloux F (2010) Discriminant analysis of principal components: a new method for the analysis of genetically structured populations. *BMC Genetics* **11**, 94.

Jombart T, Devillard S, Dufour A-B, Pontier D (2008) Revealing cryptic spatial patterns in genetic variability by a new multivariate method. *Heredity* **101**, 92-103.

Jombart T, Pontier D, Dufour A-B (2009) Genetic markers in the playground of multivariate analysis. *Heredity* **102**, 330-341.

Jones AG, Small CM, Paczolt Ka, Ratterman NL (2010) A practical guide to methods of parentage analysis. *Molecular Ecology Resources* **10**, 6-30.

Jones OR, Wang J (2010) COLONY: a program for parentage and sibship inference from multilocus genotype data. *Molecular Ecology Resources* **10**, 551-555.

Jost L (2008) *G(ST)* and its relatives do not measure differentiation. *Molecular Ecology* **17**, 4015-4026.

Jost L (2009) *D* vs. *G(ST)*: Response to Heller and Siegismund (2009) and Ryman and Leimar (2009). *Molecular Ecology* **18**, 2088-2091.

Kaeuffer R, Réale D, Coltman DW, Pontier D (2007) Detecting population structure using STRUCTURE software: effect of background linkage disequilibrium. *Heredity* **99**, 374-380.

Kalinowski ST (2009) How well do evolutionary trees describe genetic relationships among populations? *Heredity* **102**, 506-513.

Kalinowski ST (2011) The computer program STRUCTURE does not reliably identify the main genetic clusters within species: simulations and implications for human population structure. *Heredity* **106**, 625-632.

Karlin EF, Andrus RE, Boles SB, Shaw AJ (2011) One haploid parent contributes 100% of the gene pool for a widespread species in northwest North America. *Molecular Ecology* **20**, 753-767.

Knowles LL (2009) Statistical phylogeography. *Annual Review of Ecology, Evolution, and Systematics* **40**, 593-612.

Koskinen MT (2003) Individual assignment using microsatellite DNA reveals unambiguous breed identification in the domestic dog. *Animal Genetics* **34**, 297-301.

Kronholm I, Loudet O, de Meaux J (2010) Influence of mutation rate on estimators of genetic differentiation--lessons from Arabidopsis thaliana. *BMC Genetics* **11**, 33.

Kuhner MK (2006) LAMARC 2.0: maximum likelihood and Bayesian estimation of population parameters. *Bioinformatics* **22**, 768-770.

Kuhner MK (2009) Coalescent genealogy samplers: windows into population history. *Trends in Ecology & Evolution* **24**, 86-93.

Landguth EL, Cushman SA, Murphy MA, Luikart G (2010a) Relationships between migration rates and landscape resistance assessed using individual-based simulations. *Molecular Ecology Resources* **10**, 854-862.

Landguth EL, Cushman SA, Schwartz MK, McKelvey KS, Murphy M, Luikart G (2010b) Quantifying the lag time to detect barriers in landscape genetics. *Molecular Ecology* **19**, 4179-4191.

Lee C, Abdool A, Huang C-H (2009) PCA-based population structure inference with generic clustering algorithms. *BMC Bioinformatics* **10**, S73.

Leng L, Zhang D-X (2011) Measuring population differentiation using *G(ST)* or *D*? A simulation study with microsatellite DNA markers under a finite island model and nonequilibrium conditions. *Molecular Ecology* **20**, 2494-2509.

Leng L, Zhang D-X (2013) Time matters: Some interesting properties of the population differentiation measures *G(ST)* and *D* overlooked in the equilibrium perspective. *Journal of Systematics and Evolution* **51**, 44-60.

Limpiti T, Intarapanich A, Assawamakin A, Shaw PJ, Wangkumhang P, Piriyapongsa J, Ngamphiw C, Tongsima S (2011) Study of large and highly stratified population datasets by combining iterative pruning principal component analysis and structure. *BMC Bioinformatics* **12**, 255.

Liu N, Zhao H (2006) A non-parametric approach to population structure inference using multilocus genotypes. *Human Genomics* **2**, 353-364.

Lloyd MW, Campbell L, Neel MC (2013) The power to detect recent fragmentation events using genetic differentiation methods. *PLoS One* **8**, e63981.

Lowe WH, Allendorf FW (2010) What can genetics tell us about population connectivity? *Molecular Ecology* **19**, 3038-3051.

Lukoschek V, Waycott M, Keogh JS (2008) Relative information content of polymorphic microsatellites and mitochondrial DNA for inferring dispersal and population genetic structure in the olive sea snake, *Aipysurus laevis*. *Molecular Ecology* **17**, 3062-3077.

Ma J, Amos CI (2012) Principal components analysis of population admixture. *PLoS One* **7**, e40115.

Manel S, Gaggiotti OE, Waples RS (2005) Assignment methods: matching biological questions with appropriate techniques. *Trends in Ecology & Evolution* **20**, 136-142.

Manel S, Holderegger R (2013) Ten years of landscape genetics. *Trends in Ecology & Evolution* **28**, 614-621.

Masucci A, Kalampokis A, Eguíluz V, Hernández-García E (2011) Extracting directed information flow networks: an application to genetics and semantics. *Physical Review E* **83**, 026103.

McVean G (2009) A genealogical interpretation of principal components analysis. *PLoS Genetics* **5**, e1000686.

Meece JK, Anderson JL, Fisher MC, Henk DA, Sloss BL, Reed KD (2011) Population genetic structure of clinical and environmental isolates of *Blastomyces dermatitidis*, based on 27 polymorphic microsatellite markers. *Applied and Environmental Microbiology* **77**, 5123-5131.

Meirmans P (2011a) *GenoDive Help* Institute for Biodiversity and Ecosystem Dynamics, University of Amsterdam, Amsterdam.

Meirmans P (2011b) *kMeans Manual* Institute for Biodiversity and Ecosystem Dynamics, University of Amsterdam, Amsterdam.

Meirmans PG (2006) Using the AMOVA framework to estimate a standardized genetic differentiation measure. *Evolution* **60**, 2399-2402.

Meirmans PG (2012) AMOVA-based clustering of population genetic data. *The Journal of Heredity* **103**, 744-750.

Meirmans PG, Hedrick PW (2011) Assessing population structure: *F(ST)* and related measures. *Molecular Ecology Resources* **11**, 5-18.

Menozzi P, Piazza A, Cavalli-Sforza LL (1978) Synthetic maps of human gene frequencies in Europeans. *Science* **201**, 786-792.

Michalakis Y, Excoffier L (1996) A generic estimation of population subdivision using distanced between alleles with special reference for microsatellite loci. *Genetics* **142**, 1061-1064.

Milligan GW, Cooper MC (1985) An examination of procedures for determining the number of clusters in a data set. *Psychometrika* **50**, 159-179.

Mimaroglu S, Aksehirli E (2011) DICLENS: Divisive Clustering Ensemble with Automatic Cluster Number. *IEEE/ACM Transactions on Computational Biology and Bioinformatics* **9**, 408-420.

Morris JH, Apeltsin L, Newman AM, Baumbach J, Wittkop T, Su G, Bader GD, Ferrin TE (2011) clusterMaker: a multi-algorithm clustering plugin for Cytoscape. *BMC Bioinformatics* **12**, 436.

Nauta MJ, Weissing FJ (1996) Constraints on allele size at microsatellite loci: implications for genetic differentiation. *Genetics* **143**, 1021-1032.

Nei M (1973) Analysis of gene diversity in subdivided populations. *Proceedings of the National Academy of Sciences* **70**, 3321-3323.

Nielsen R, Beaumont MA (2009) Statistical inferences in phylogeography. *Molecular Ecology* **18**, 1034-1047.

Nielsen R, Wakeley J (2001) Distinguishing migration from isolation: a Markov chain Monte Carlo approach. *Genetics* **158**, 885-896.

Novembre J, Stephens M (2008) Interpreting principal component analyses of spatial population genetic variation. *Nature Genetics* **40**, 646-649.

Odong TL, van Heerwaarden J, Jansen J, van Hintum TJL, van Eeuwijk FA (2011) Determination of genetic structure of germplasm collections: are traditional hierarchical clustering methods appropriate for molecular marker data? *Theoretical and Applied Genetics* **123**, 195-205.

Onogi A, Nurimoto M, Morita M (2011) Characterization of a Bayesian genetic clustering algorithm based on a Dirichlet process prior and comparison among Bayesian clustering methods. *BMC Bioinformatics* **12**, 263.

Palczewski M, Beerli P (2013) A continuous method for gene flow. *Genetics* **194**, 687-696.

Palsbøll PJ, Zachariah Peery M, Bérubé M (2010) Detecting populations in the 'ambiguous' zone: kinship-based estimation of population structure at low genetic divergence. *Molecular Ecology Resources* **10**, 797-805.

Patterson N, Price AL, Reich D (2006) Population structure and eigenanalysis. *PLoS Genetics* **2**, e190.

Peakall R, Smouse PE (2012) GenAlEx 6.5: genetic analysis in Excel. Population genetic software for teaching and research–an update. *Bioinformatics* **28**, 2537-2539.

Pearson K (1901) On lines and planes of closest fit to systems of points in space. *Philosophical Magazine* **2**, 559-572.

Pella J, Masuda M (2006) The Gibbs and split–merge sampler for population mixture analysis from genetic data with incomplete baselines. *Canadian Journal of Fisheries and Aquatic Sciences* **63**, 576-596.

Peter BM, Wegmann D, Excoffier L (2010) Distinguishing between population bottleneck and population subdivision by a Bayesian model choice procedure. *Molecular Ecology* **19**, 4648-4660.

Pinho C, Hey J (2010) Divergence with gene flow: models and data. *Annual Review of Ecology, Evolution, and Systematics* **41**, 215-230.

Porras-Hurtado L, Ruiz Y, Santos C, Phillips C, Carracedo A, Lareu MV (2013) An overview of STRUCTURE: applications, parameter settings, and supporting software. *Frontiers in Genetics* **4**, 98.

Pritchard JK, Stephens M, Donnelly P (2000) Inference of population structure using multilocus genotype data. *Genetics* **155**, 945-959.

Pritchard JK, Wen X, Falush D (2010) *Documentation for structure software* Department of Human Genetics, University of Chicago, Chicago.

Rajaram S, Oono Y (2010) NeatMap—non-clustering heat map alternatives in R. *BMC Bioinformatics* **11**, 45.

Reeves PA, Richards CM (2009) Accurate inference of subtle population structure (and other genetic discontinuities) using principal coordinates. *PLoS One* **4**, e4269.

Robert CP, Cornuet J-M, Marin J-M, Pillai NS (2011) Lack of confidence in approximate Bayesian computation model choice. *Proceedings of the National Academy of Sciences* **108**, 15112-15117.

Rodríguez-Ramilo ST, Wang J (2012) The effect of close relatives on unsupervised Bayesian clustering algorithms in population genetic structure analysis. *Molecular Ecology Resources* **12**, 873-884.

Rosenberg NA, Pritchard JK, Weber JL, Cann HM, Kidd KK, Zhivotovsky LA, Feldman MW (2002) Genetic structure of human populations. *Science* **298**, 2381-2385.

Rossetto M, Thurlby KA, Offord CA, Allen CB, Weston PH (2011) The impact of distance and a shifting temperature gradient on genetic connectivity across a heterogeneous landscape. *BMC Evolutionary Biology* **11**, 126.

Rousset F (1996) Equilibrium values of measures of population subdivision for stepwise mutation processes. *Genetics* **142**, 1357-1362.

Rousset F (2013) Exegeses on maximum genetic differentiation. *Genetics* **194**, 557-559.

Ryman N, Leimar O (2008) Effect of mutation on genetic differentiation among nonequilibrium populations. *Evolution* **62**, 2250-2259.

Ryman N, Leimar O (2009) *G(ST)* is still a useful measure of genetic differentiation—a comment on Jost’s *D*. *Molecular Ecology* **18**, 2084-2087.

Safner T, Miller MP, McRae BH, Fortin M-J, Manel S (2011) Comparison of bayesian clustering and edge detection methods for inferring boundaries in landscape genetics. *International Journal of Molecular Sciences* **12**, 865-889.

Saisho D, Purugganan MD (2007) Molecular phylogeography of domesticated barley traces expansion of agriculture in the Old World. *Genetics* **177**, 1765-1776.

Schwartz MK, McKelvey KS (2008) Why sampling scheme matters: the effect of sampling scheme on landscape genetic results. *Conservation Genetics* **10**, 441-452.

Sefc KM, Payne RB, Sorenson MD (2007) Genetic differentiation after founder events: an evaluation of *F(ST)* estimators with empirical and simulated data. *Evolutionary Ecology Research* **9**, 21-39.

Segelbacher G, Cushman SA, Epperson BK, Fortin M-J, Francois O, Hardy OJ, Holderegger R, Taberlet P, Waits LP, Manel S (2010) Applications of landscape genetics in conservation biology: concepts and challenges. *Conservation Genetics* **11**, 375-385.

Shannon CE (1948a) A mathematical theory of communication. *Bell System Technical Journal* **27**, 379-423.

Shannon CE (1948b) A mathematical theory of communication. *Bell System Technical Journal* **27**, 623-656.

Sherwin WB (2010) Entropy and information approaches to genetic diversity and its expression: genomic geography. *Entropy* **12**, 1765-1798.

Sherwin WB, Jabot F, Rush R, Rossetto M (2006) Measurement of biological information with applications from genes to landscapes. *Molecular Ecology* **15**, 2857-2869.

Shringarpure S, Won D, Xing EP (2011) StructHDP: automatic inference of number of clusters and population structure from admixed genotype data. *Bioinformatics* **27**, i324-i332.

Slatkin M (1993) Isolation by distance in equilibrium and non-equilibrium populations. *Evolution* **47**, 264-279.

Slatkin M (1995) A measure of population subdivision based on microsatellite allele frequencies. *Genetics* **139**, 457-462.

Sodhi M, Mukesh M, Ahlawat SPS, Sobti RC, Gahlot GC, Mehta SC, Prakash B, Mishra BP (2008) Genetic diversity and structure of two prominent zebu cattle breeds adapted to the arid region of India inferred from microsatellite polymorphism. *Biochemical Genetics* **46**, 124-136.

Sokal R, Michener C (1958) A statistical method for evaluating systematic relationships. *University of Kansas Science Bulletin* **38**, 1409-1438.

Song S, Dey DK, Holsinger KE (2006) Differentiation among populations with migration, mutation and drift: implications for genetic inference. *Evolution* **60**, 1-12.

Song S, Dey DK, Holsinger KE (2011) Genetic diversity of microsatellite loci in hierarchically structured populations. *Theoretical Population Biology* **80**, 29-37.

Sousa VC, Beaumont MA, Fernandes P, Coelho MM, Chikhi L (2012) Population divergence with or without admixture: selecting models using an ABC approach. *Heredity* **108**, 521-530.

Sousa VC, Carneiro M, Ferrand N, Hey J (2013) Identifying loci under selection against gene flow in isolation-with-migration models. *Genetics* **194**, 211-233.

Sousa VC, Fritz M, Beaumont MA, Chikhi L (2009) Approximate Bayesian computation without summary statistics: the case of admixture. *Genetics* **181**, 1507-1519.

Storfer A, Murphy MA, Evans JS, Goldberg CS, Robinson S, Spear SF, Dezzani R, Delmelle E, Vierling L, Waits LP (2007) Putting the "landscape" in landscape genetics. *Heredity* **98**, 128-142.

Sun JX, Mullikin JC, Patterson N, Reich DE (2009) Microsatellites are molecular clocks that support accurate inferences about history. *Molecular Biology and Evolution* **26**, 1017-1027.

Sunnåker M, Busetto AG, Numminen E, Corander J, Foll M, Dessimoz C (2013) Approximate Bayesian computation. *PLoS Computational Biology* **9**, e1002803.

Takezaki N, Nei M (1996) Genetic distances and reconstruction of phylogenetic trees from microsatellite DNA. *Genetics* **144**, 389-399.

Thalamuthu A, Mukhopadhyay I, Zheng X, Tseng GC (2006) Evaluation and comparison of gene clustering methods in microarray analysis. *Bioinformatics* **22**, 2405-2412.

Wadl PA, Wang X, Trigiano AN, Skinner JA, Windham MT, Trigiano RN, Rinehart TA, Reed SM, Pantalone VR (2008) Molecular identification keys for cultivars and lines of *Cornus florida* and *C. kousa* based on simple sequence repeat loci. *Journal of the American Society for Horticultural Science* **133**, 783-793.

Wahlund S (1928) Composition of populations and correlation appearances viewed in relation to the studies of inheritance. *Hereditas* **11**, 65-106.

Wang J (2004) Sibship reconstruction from genetic data with typing errors. *Genetics* **166**, 1963-1979.

Wang J (2011) COANCESTRY: a program for simulating, estimating and analysing relatedness and inbreeding coefficients. *Molecular Ecology Resources* **11**, 141-145.

Wang J (2012a) Computationally efficient sibship and parentage assignment from multilocus marker data. *Genetics* **191**, 183-194.

Wang J (2012b) On the measurements of genetic differentiation among populations. *Genetics Research* **94**, 275-289.

Waples RS, Gaggiotti O (2006) What is a population? An empirical evaluation of some genetic methods for identifying the number of gene pools and their degree of connectivity. *Molecular Ecology* **15**, 1419-1439.

Waples RS, Waples RK (2011) Inbreeding effective population size and parentage analysis without parents. *Molecular ecology resources* **11**, 162-171.

Ward JH (1963) Hierarchical grouping to optimize an objective function. *Journal of the American Statistical Association* **58**, 236-244.

Weir BS, Cockerham CC (1984) Estimating *F*-statistics for the analysis of population structure. *Evolution* **38**, 1358-1370.

Weir BS, Hill WG (2002) Estimating *F*-statistics. *Annual Review Genetics* **36**, 721-750.

Whitlock MC (2011) *G'(ST)* and *D* do not replace *F(ST)*. *Molecular Ecology* **20**, 1083-1091.

Whitlock MC, McCauley DE (1999) Indirect measures of gene flow and migration: *F(ST)* not equal to 1/(4*Nm* + 1). *Heredity* **82**, 117-125.

Wright S (1943) Isolation by distance. *Genetics* **28**, 114-138.

Wright S (1978) *Evolution and the Genetics of Populations, Volume 4: Variability Within and Among Natural Populations* University of Chicago Press, Chicago, Illinois.

Wu C-H, Drummond AJ (2011) Joint inference of microsatellite mutation models, population history and genealogies using transdimensional Markov Chain Monte Carlo. *Genetics* **188**, 151-164.

Xu R, Wunsch D (2005) Survey of clustering algorithms. *IEEE Transactions on Neural Networks* **16**, 645-678.

Zhivotovsky LA, Rosenberg NA, Feldman MW (2003) Features of evolution and expansion of modern humans, inferred from genomewide microsatellite markers. *American Journal of Human Genetics* **72**, 1171-1186.
